# Protocol for mapping murine myeloid bone marrow progenitors and their differentiation into CD103^+^ cDC1s and CD301b^+^ cDC2s

**DOI:** 10.1016/j.xpro.2026.104508

**Published:** 2026-05-02

**Authors:** Damir Vurnek, Giorgi Tchitashvili, Anna Seichter, Tomislav Vuletic, Isabel Heß, Lizi Trapaidze, Lukas Heger, Nounagnon Romaric Tochoedo, Christian H.K. Lehmann, Bart N. Lambrecht, Diana Dudziak, Lukas Amon

**Affiliations:** 1 Institute of Immunology, Jena University Hospital, Friedrich-Schiller-University Jena, 07743 Jena, Germany; 2 Laboratory of Dendritic Cell Biology, Department of Dermatology, University Hospital, Friedrich-Alexander University of Erlangen-Nürnberg, 91052 Erlangen, Germany; 3 Department of Gastroenterology and Hepatology, University Hospital Zurich, University of Zurich, 8091 Zurich, Switzerland; 4 Institute of Physics, 10000 Zagreb, Croatia; 5 VIB, Suzanne Tassierstraat 1, 9052 Gent, Belgium; 6 Department of Transfusion Medicine and Hemostaseology, University Hospital Erlangen, Friedrich-Alexander University of Erlangen-Nürnberg, 91054 Erlangen, Germany; 7 Department of Pediatrics and Adolescent Medicine, Friedrich-Alexander-University (FAU) Erlangen-Nürnberg, 91054 Erlangen, Germany; 8 FAU Profile Center Immunomedicine (FAU I-MED), 91054 Erlangen, Germany; 9 Laboratory of Immunoregulation and Mucosal Immunology, VIB Center for Inflammation Research, 9052 Ghent, Belgium; 10 Department of Internal Medicine and Pediatrics, Ghent University, 9000 Ghent, Belgium; 11 Department of Pulmonary Medicine, Erasmus University Medical Center Rotterdam, 3015 Rotterdam, the Netherlands; 12 Cluster of Excellence Balance of the Microverse, Friedrich Schiller University Jena, 07743 Jena, Germany; 13 Center for Sepsis Control and Care, Jena University Hospital, 07747 Jena, Germany; 14 Zentrum für Alternsforschung Jena (ZAJ), Jena, Germany; 15 Comprehensive Cancer Center Central Germany (CCCG), 07743 Jena, Germany; 16 Core Facility Cytometry, Jena University Hospital, 07743 Jena, Germany

**Keywords:** Cell culture, Cell isolation, Cell-based Assays, Flow Cytometry, Immunology, Cell Differentiation

## Abstract

*Ex vivo* differentiation platforms have a long-standing history for studying dendritic cell (DC) ontogeny and function. Here, we present a protocol for differentiating *bona fide* DCs from murine bone marrow. We describe steps for bone marrow preparation, followed by *ex vivo* differentiation of DC subtypes resembling either lymphoid or mucosal tissue DC phenotypes. We then detail procedures for flow cytometric analysis and sorting strategies that facilitate phenotypic and functional studies of murine DC subtypes and/or their bone marrow progenitors.

For complete details on the use and execution of this protocol, please refer to Amon et al.^1^

## Highlights

• *Ex vivo* differentiation of *bona fide* DCs from murine bone marrow
• FLT3L priming followed by delayed GM-CSF supplementation
• Generation of cDC1s and cDC2s with CD103 or CD301b expression
• Flow cytometric profiling of myeloid progenitors and DC subsets

## Abstract (editor highlights)

Publisher’s note: Undertaking any experimental protocol requires adherence to local institutional guidelines for laboratory safety and ethics.

## Graphical abstract

**Figure:**
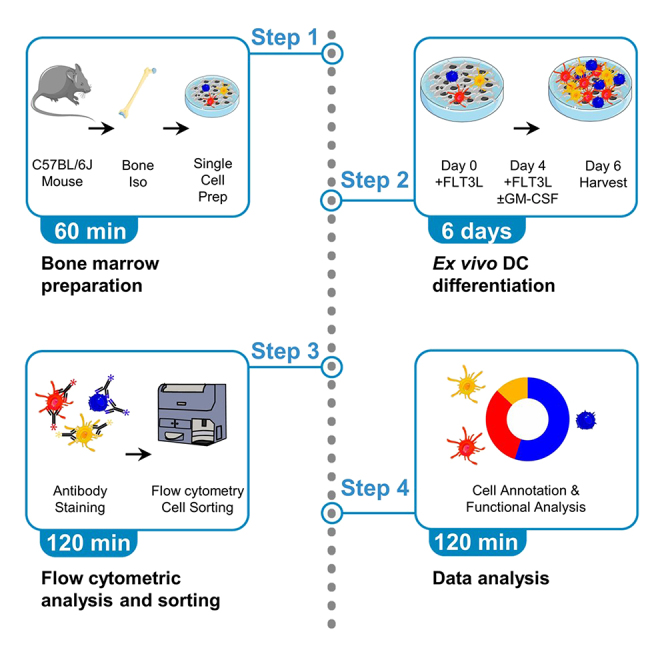

## Before you begin

This protocol describes the preparation of single cell suspensions from murine bone marrow, the subsequent differentiation of *bona fide* DC subtypes, and their phenotypic analysis via flow cytometry. However, bone marrow single cell suspensions or DC subsets sorted post differentiation can be used within additional downstream applications including functional assays (antigen presentation assays, cytokine measurements, migration, cell transfers) or sequencing procedures.^2^^,^^3^ The following protocol utilizes Lympholyte-M Cell Separation Media for erythrocyte removal. Before starting, bring Lympholyte-M and FBS-free RPMI-1640 to 18**–**22°C. If Lympholyte-M is not available, gradient centrifugation can be substituted with osmotic lysis reagents. Ensure that the flow cytometer and/or sorter are able to excite, detect, and separate fluorescence signals from utilized fluorophores. Check that all antigens are expressed in the mouse strains used (such as MHC-II alloantigens).

### Innovation

Vast efforts in establishing granulocyte-macrophage colony-stimulating factor (GM-CSF) cultures conducted by the DC community have tremendously advanced our understanding of murine and human DC biology and enabled the development of DC-based therapeutic approaches, including autologous DC transfers.^4^^,^^5^^,^^6^^,^^7^^,^^8^^,^^9^^,^^10^^,^^11^^,^^12^^,^^13^^,^^14^ However, many published protocols for DC generation from bone marrow utilizing GM-CSF alone or in combination with other cytokines (interleukin-4, IL-4; Fms-like tyrosine kinase 3 ligand, FLT3L) relied on the addition of GM-CSF at the start of *ex vivo* cultures. This likely promoted the abundant generation of monocyte-derived DCs (moDCs) and macrophages (moMACs).^7^ We here provide a protocol that circumvents moDC and moMAC generation by the sequential application of FLT3L and GM-CSF leading to the generation of *bona fide* DC subsets. In cultures only containing FLT3L, conventional DCs type 1 (cDC1s) and type 2 (cDC2s) are generated that display a classic lymphoid tissue phenotype. Moreover, the timed addition of GM-CSF allows for the generation of cDC1s and cDC2s characterized by CD103 and CD301b expression, respectively.^1^ *In vivo*, DCs of these phenotypes are preferentially found in mucosal tissues.^3^^,^^4^^,^^15^^,^^16^^,^^17^^,^^18^^,^^19^^,^^20^^,^^21^ Hence, our protocol enables the generation of DC subsets displaying either lymphoid or mucosal tissue phenotypes.

### Institutional permissions

Prior to experimenting, acquire all necessary permits to perform vertebrate experiments in accordance to your institutional and national guidelines. Experiments described herein were compliant with institutional and national guidelines and approved by the Amt für Veterinärwesen und gesundheitlichen Verbraucherschutz (TS-6/12; TS-11/2022). Female C57BL/6 J mice (analyzed between 8 and 15 weeks of age) were bought from Charles River. Mice were housed in the Präklinisches Experimentelles Tierzentrum (Friedrich-Alexander-Universität Erlangen-Nürnberg) under specific pathogen-free conditions consistent with FELASA guidelines.

## Key resources table

**Table:** 

REAGENT or RESOURCE	SOURCE	IDENTIFIER
**Antibodies**		

anti-mouse CD172a PerCP-e710 (Clone P84; 1:400-1:800)	eBioscience (Thermo Fisher Scientific)	#46-1721-82; RRID: AB_10804639
anti-mouse/human CD11b A700 (Clone M1/70; 1:400)	BioLegend	#101222; RRID: AB_493705
anti-mouse CD88 APC (Clone 20/70; 1:100)	BioLegend	#135808; RRID: AB_10899415
anti-mouse CD103 APC-Cy7 (Clone 2E7; 1:100)	BioLegend	#121432; RRID: AB_2566551
anti-mouse CD117 APC-Fire750 (Clone 2B8; 1:400)	BioLegend	#105838; RRID: AB_2616739
anti-mouse Ly6C APC-e780 (Clone HK1.4; 1:800)	eBioscience (Thermo Fisher Scientific)	#47-5932-82; RRID: AB_2573992
anti-mouse NK1.1 BUV395 (Clone PK136; 1:400)	BD Biosciences	#564144; RRID: AB_2738618
anti-mouse CD3ε BUV737 (Clone 17A2; 1:200)	BD Biosciences	#612803; RRID: AB_2870130
anti-mouse CD19 BUV737 (Clone 1D3; 1:400)	BD Biosciences	#612781; RRID: AB_2870110
anti-mouse Siglec-H BUV737 (Clone 440c; 1:200)	BD Biosciences	#748293; RRID: AB_2872719
anti-mouse Siglec-H FITC (Clone 551; 1:100)	BioLegend	#129604; RRID: AB_1227760
anti-mouse/rat XCR1 BV421 (Clone ZET; 1:200)	BioLegend	#148216; RRID: AB_2565230
anti-mouse/rat XCR1 PE (Clone ZET; 1:400)	BioLegend	#148204; RRID: AB_2563843
anti-mouse CD34 BV421 (Clone SA376A4; 1:200)	BioLegend	#152208; RRID: AB_2650766
anti-mouse Ly-6C BV570 (Clone HK1.4; 1:400-1:800)	BioLegend	#128030; RRID: AB_2562617
anti-mouse CD45 BV605 (Clone 30-F11; 1:400)	BioLegend	#103140; RRID: AB_2562341
anti-mouse CD135 PE (Clone A2F10; 1:200)	eBioscience (Thermo Fisher Scientific)	#12-1351-82; RRID: AB_465859
anti-mouse CD172a AF488 (Clone P84; 1:800)	BioLegend	#144024; RRID: AB_2650815
anti-mouse CD11c PE-CF594 (Clone HL3; 1:400-1:800)	BD Biosciences	#562454; RRID: AB_2737617
anti-mouse/human B220 PE-Cy5 (Clone RA3-6B2; 1:800)	BioLegend	#103210; RRID: AB_312995
anti-mouse TCRbeta PE-Cy5 (Clone H57-597; 1:2000)	eBioscience (Thermo Fisher Scientific)	#15-5961-82; RRID: AB_468816
anti-mouse CD49b PE-Cy5 (Clone DX5; 1:200)	eBioscience (Thermo Fisher Scientific)	#15-5971-82; RRID: AB_2573070
anti-mouse CD301b PE-Cy7 (Clone URA-1; 1:1000-1:2000)	BioLegend	#146808; RRID: AB_2563390
anti-mouse CD115 PE-Cy7 (Clone AFS98; 1:200)	BioLegend	#135524; RRID: AB_2566460
anti-mouse Ly-6A/E (Sca-1) PerCP-Cy5.5 (Clone E13–161.7; 1:200)	BioLegend	#122524; RRID: AB_893617
anti-mouse MHC-II (I-A & I-E) V500 (Clone M5/114.15.2; 1:200)	BD Biosciences	#562366; RRID: AB_11153488
anti-mouse CD16/CD32 (FcγRIIB/III) BV650 (Clone AB93; 1:200-1:800)	BD Biosciences	#751690; RRID: AB_2875675
anti-mouse CD3ε Biotin (Clone 145-2C11; 1:200-1:400)	BioLegend	#100304; RRID: AB_312669
anti-mouse CD19 Biotin (Clone 6D5; 1:400)	BioLegend	#115504; RRID: AB_313638
anti-mouse Ly-6G Biotin (Clone 1A8; 1:400)	BioLegend	#127604; RRID: AB_1186105
anti-mouse Ter119 Biotin (Clone TER-119; 1:800)	BioLegend	#116204; RRID: AB_313704
Purified anti-mouse CD16/32 (FcγRIIB/III) (Clone 2.4G2; 1:400)	BioXCell	#BE0307; RRID: AB_2736987
Purified anti-mouse CD16.2 (FcγRIV) (Clone 9E9; 1:400)	BioLegend	#149502; RRID: AB_2565302
Streptavidin BUV496 (1:50)	BD Biosciences	#612961; RRID: AB_2870237

**Chemicals, peptides, and recombinant proteins**		

HEPES buffer solution (1 M)	Gibco	#15630-056
β-Mercaptoethanol 50 mM in DPBS	PAN Biotech	#P07-05100
L-glutamine solution	Sigma Aldrich	#G7513
Penicillin-Streptomycin solution	Sigma Aldrich	#P4333
EDTA UltraPure 0.5 M, pH 8.0	Invitrogen	#15575-038
Lympholyte-M	Cedarlane	#CL5035
Recombinant mouse FMS-like tyrosine kinase 3 ligand (FLT3-L; carrier-free)	BioLegend	#550706
Mouse Granulocyte-macrophage colony-stimulating-factor (GM-CSF)	PeproTech Inc.	#315-03
Acetic acid	Roth	#6755.1
Ammonium chloride (NH_4_Cl)	Roth	#K298.1
Potassium hydrogen carbonate (KHCO_3_)	Roth	#P748.1
Dulbecco’s Phosphate Buffered Saline without calcium and magnesium (DPBS)	Sigma	#D8537
RPMI-1640 (with phenol red)	Sigma	#R8758
RPMI-1640 (without phenol red)	Gibco	#11835-063
Fetal Bovine Serum (FBS)	Sigma	#F7524
4′,6-diamidino-2-phenylindole (DAPI)	AppliChem	#A4099
Water BioPerformance Certified	Sigma	#W3513

**Experimental models: Organisms/strains**		

C57BL/6 J wildtype mice (female, between 8-15 weeks of age)	Charles River	#C57/BL6″J″

**Software and algorithms**		

DIVA V7.0	BD Biosciences	https://www.bdbiosciences.com/en-de/products/software/instrument-software/bd-facsdiva-software
FlowJo V10	BD Biosciences	https://www.bdbiosciences.com/en-de/products/software/flowjo-v10-software
Plugin UMAP V4.0.3	McInnes et al., ArXiv, 2019 (https://doi.org/10.48550/arXiv.1802.03426)	https://www.flowjo.com/exchange/#/plugin/profile?id=6
Plugin down sample V3.3.1	BD Biosciences	https://www.flowjo.com/exchange/#/plugin/profile?id=25
Office 2019	Microsoft	https://www.microsoft.com/de-de/
CorelDRAW 2021	Alludo	https://www.coreldraw.com

**Other**		

Flow cytometer	BD Biosciences	BD LSRFortessa SORP
Cell sorter	BD Biosciences	BD Aria II SORP
Sterile work bench	Scanlaf	Mars Safety Class 2
Light-microscope	Nikon	Eclipse TS100
Incubator “HERAEUS BBD6220”	Thermo Scientific	#51020241
Centrifuge “Allegra X-15 R″	Beckman-Coulter	#392934
Neubauer chamber 0.100 mm; 0.0025 mm^2^	Superior Marienfeld	#0640010
Diaphragm vacuum pump	vacuubrand	#MZ 2C+AK+EK
Water bath “Type 1086”	GFL	#10237998 C
100 μm cell strainers	Greiner bio-one	#542000
40 μm cell strainers	Greiner bio-one	#542140
Pestles	BD Biosciences	#309658
Sterile storage bottles	Corning	#430518
0.22 μm sterile filters	Corning	#431118
6-well plates	Greiner bio-one	#657160
96-well V-bottom plates	Greiner bio-one	#651101
Tissue forceps	Carl Teufel	#2400-10
Dressing forceps	Carl Teufel	#2410-10
Scissors	Carl Teufel	#4704-01
27-gauge needles	Braun Sterican	#4657519 B
23-gauge needles	Braun Sterican	#4657667 B
Discardit II 10 mL syringe	BD Biosciences	#309110
5 mL polystyrene round-bottom tube 12x75 mm style	Corning Falcon®	#352008
5 mL polystyrene round-bottom low binding tube with Snap Cap	Corning Falcon®	#352063
5 mL polystyrene round-bottom tube with Cell Strainer Snap Cap	Corning Falcon®	#352235
FACSFlow Sheath Fluid	BD Biosciences	#342003
UltraComp eBeads Plus Compensation Beads	eBioscience (Thermo Fisher Scientific)	#01-3333-42
Precision Count Beads™	BioLegend	#424902
BD FACSDiva™ CS&T Research Beads	BD Biosciences	#655050
BD FACS™ Accudrop Beads	BD Biosciences	#345249

## Materials and equipment

Prepare all buffers and stock solutions under aseptic conditions using sterile reagents, solutions, and consumables.

### Fetal bovine serum

Quickly thaw FBS at 37°C in a water bath. After thawing, incubate FBS for 30 min (minutes) at 42°C to inactivate complement components. Immediately pass the warmed FBS through a 0.22 μm filter into a storage bottle inside the sterile hood. Dispense into 50 mL aliquots, store at −20°C, and refrain from freeze-thaw cycles. Before using a new FBS lot, evaluate multiple candidates. Whenever possible, benchmark the new lots against the currently used one by examining cellular yield and activation marker profile of mouse bone marrow-derived DCs after differentiation. Only use a lot that reproduces both, the cellular output and activation phenotype of the existing lot.

### RPMI + 2% FBS

Add 2% (v/v) FBS to RPMI-1640 solution and store at 4°C until use. The medium can be used repeatedly, if sterility is preserved.

### DC differentiation medium

Supplement RPMI-1640 solution without indicator (phenol red) with 10% (v/v) FBS, 2 mM L-Glutamine, 100 U/mL penicillin, 100 μg/mL streptomycin, 50 μM β-mercaptoethanol, and 10 mM 4-(2-hydroxyethyl)-1-piperazineethanesulfonic acid (HEPES) and store at 4°C until use. The medium can be used repeatedly, if sterility is preserved. For long term cultures, we recommend to use phenol red-free RPMI-1640 medium, since it is known that phenol red can mimic estrogen stimulation potentially acting on DC-poiesis.

### FACS buffer

Supplement phosphate-buffered saline solution (PBS) with 2% (v/v) FBS and store at 4°C until use. The medium can be used repeatedly, if sterility is preserved.

### Sort buffer

Supplement FACS buffer with 2 mM Ethylenediaminetetraacetic acid (EDTA) and store at 4°C until use. The medium can be used repeatedly, if sterility is preserved.

**Table:** Ammonium-Chloride-Potassium (ACK) lysis buffer

Reagent	Final concentration	Amount
NH_4_Cl	155 mM	15.5 mmol
KHCO_3_	10 mM	1 mmol
double-distilled H_2_O	N/A	–
HCl	adjust pH to 7.23	–
**Total**	–	**100 mL**

Simultaneously dissolve ammonium chloride (NH_4_Cl) and potassium hydrogen carbonate (KHCO_3_) in double-distilled water to final concentrations of 155 mM and 10 mM, respectively. Adjust the pH to 7.23 with HCl, if necessary. Store the autoclaved solution at 20**–**22°C. Once opened, do not use the ACK lysis buffer for longer than six weeks, as airborne CO_2_ alters the pH. Commercially prepared ACK lysis buffer can be used analogously.

### DAPI stock solution

Dissolve lyophilized 4′,6-diamidino-2-phenylindole dihydrochloride (DAPI) in ultrapure water to obtain a final concentration of 1 mg/mL. Store the solution at 4°C in the dark for up to six months.

### DAPI working solution

Dilute the DAPI stock solution in FACS buffer by a factor of 1:10,000 to a final concentration of 100 ng/mL. Prepare the DAPI working solution always fresh.

### Acetic acid working solution

Carefully dilute 100% acetic acid solution 1:874 with ultrapure water to a working concentration of 20 mM. Freeze aliquots of 10 μL at −80°C together with GM-CSF stock vials. Acetic acid aliquots could be stored at 18**–**22°C. However, we recommend to handle the acetic acid used for controls identically to GM-CSF solutions prepared in acetic acid.

### GM-CSF stock

Dissolve lyophilized GM-CSF in 20 mM acetic acid solution according to the manufacturer’s instructions to a final concentration of 500 IU/μL. Store aliquots at −80°C until use. GM-CSF can be used until its expiration date as indicated by the manufacturer. Refrain from freeze-thaw cycles.

### FLT3L stock

The stock solution is provided ready-to-use by the manufacturer. Upon first usage, make aliquots and store them at −80°C. FLT3L can be used until its expiration date as indicated by the manufacturer. Refrain from freeze-thaw cycles.

## Step-by-step method details

### Bone isolation

**Timing: 5–10 min per mouse**

This step describes the harvesting of murine tibias and femurs.

1. Euthanize a mouse according to your license.
  a. Perform cranial dislocation.
  b. Check reflexes to ensure terminal euthanasia.
  c. Position the mouse on the preparation table with its back facing down (ventral side up).
  d. Extend legs sequentially in a distal direction and fix the mouse to the preparation table by puncturing each paw with a 23-gauge needle (Figure 1A).

**Figure 1:**
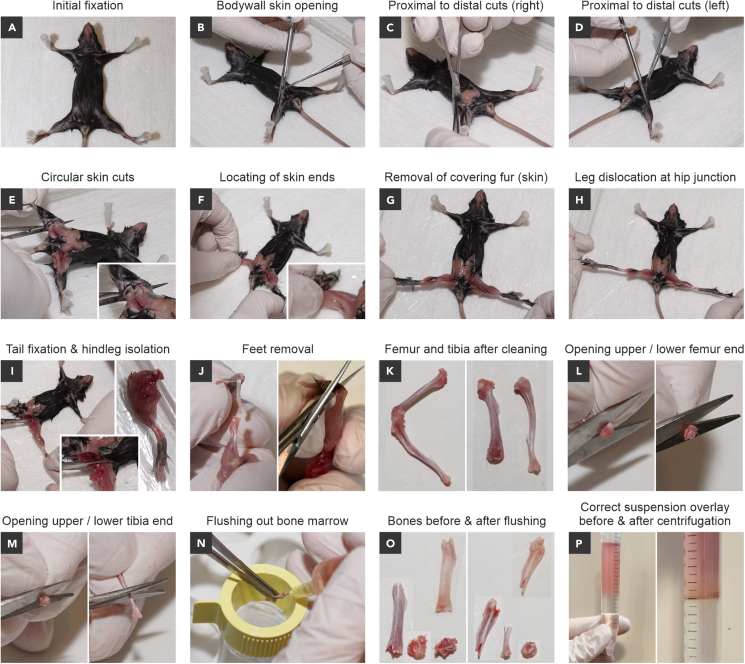
From mouse preparation to single cell bone marrow cell suspension (A) Fixation of a euthanized C57BL/6 J mouse to the preparation table. (B) Opening of the abdominal wall. (C and D) Proximal to distal cuts in the direction of the extremities. (E–G) Removal of leg-covering skin and fur. (H) Hip dislocation. (I) Left: Isolation of hindlegs. Bottom: Close-up of the incision. Right: Isolated leg. (J) Left: Feet removal by rotating and pulling. Right: Feet removal by cutting at the ankle joint. (K) Reference picture of connected (left) and disconnected (right) tibia and femur after tissue removal and bone cleaning. (L) Opening of femurs at both ends to allow for flushing out bone marrow. (M) Opening of tibias to allow for flushing out bone marrow. (N) Bone marrow isolation. (O) Reference example of femur (left) and tibia (right). Before flushing (below) and after flushing of bone marrow (above). (P) Left: Correct overlay of the cell suspension on top of Lympholyte-M, without mixing. Right: Lymphocytes collected at the interface after gradient centrifugation.
2. Isolate tibias and femurs of hindlegs.
  a. Fix hindlegs to the preparation table.
  b. Cut the body wall skin.
  c. Open using scissors on the ventral side.
  d. Perform cuts from proximal to distal direction of the extremities (Figures 1B–1D).
  e. Remove fixing needles for hindlegs.
  f. Cut the skin in a circular fashion at the femur-hip junction at both hindlegs (Figure 1E).
  g. Grab the skin covering the hindlegs close to the circular incision (Figure 1F) and pull it from proximal to distal in the direction of the extremities (Figure 1G).
  h. Dislocate femurs from the hip joint by pulling the legs laterally away from the sagittal axis (Figure 1H).
    **CRITICAL:** Dislocation of femurs from hip joints facilitates preservation of femur integrity during leg isolation. In this way, chances of severing the femoral heads during cutting are minimized.
  i. Fix the mouse tail and use the scissors free hand to stretch the processed leg away from the hip. (Figure 1I, left).
  j. Carefully cut-off hindlegs (Figure 1I, insert down).
  k. Store the complete leg (Figure 1I, right) in an empty 6-well plate on ice until all mice are processed.
    ***Note:*** During cutting, gently probe the leg with the scissors. If resistance is felt, stop immediately and adjust the scissors position and cutting site. If the femur has been properly dislocated from the hip joint, the hindleg can be cut without resistance. In rare cases the femur head will be visible even before the cut is fully executed (Figure 1I, insert down). Memorizing such reference resistance makes it easier to adjust the scissors position in future experiments. In the case no resistance-free cutting area can be identified, repeat the hip dislocation on that leg.
  l. Remove feet by rotating them against their natural moving direction (Figure 1J, left) leading to tibia exposure.
    ***Note:*** By pulling the foot away, fibula is usually removed together with the foot (requires practice). Alternatively, cut off feet using scissors (Figure 1J, right) and remove the muscles and fibulas during subsequent bone cleaning procedures.
  m. Isolate tibias, remove fibulas, and store on ice until further processing.
  n. Remove muscles and tissue from femurs by cutting parallel to the bone without rupturing it.
  o. If feet were removed with scissors do the same for the lower part of the leg.
  p. Remove residual tissue by rubbing with a gauze bandage or alternatively rugged paper tissue wipes.
    ***Note:*** Rub along the bone as pressure executed perpendicularly might inadvertently cause the bone to break.
  q. Store cleaned femurs and tibias from the same donor (Figure 1K) in empty 6-well plates on ice until all mice are processed
  r. Proceed immediately to the preparation of bone marrow single cell suspensions.

### Preparation of bone marrow single-cell suspensions

**Timing: ACK lysis variant, 30 min one mouse + 8 min per additional mouse; gradient variant, 60 min one mouse + 13 min per additional mouse**

This step describes the preparation of bone marrow single cell suspensions.

***Note:*** During bone marrow single cell preparation, either ACK lysis or gradient centrifugation will be used. While erythrocyte removal via ACK lysis is fast, it is harsh to cells and fails to deplete granulocytes. Therefore, ACK lysis is only used if generated single cell suspensions are subject to direct staining and subsequent flow cytometric analysis. If cell suspensions are used for downstream sorting and differentiation procedures, we recommend gradient centrifugation for erythrocyte removal. While it is slower than ACK lysis, it is milder to cells and reduces the percentage of granulocytes, which can be detrimental within differentiation cultures. If not specifically stated otherwise, all centrifugations are performed at 700 x *g* for 5 min at 4°C with the rotor acceleration and brake set to maximum.

3. Flush out bone marrow from tibias and femurs.
  a. Sequentially dip tibias and femurs for 4**–**5 s into 70% ethanol (EtOH) followed by washing in PBS.
    **CRITICAL:** If bones were ruptured or damaged during the isolation process omit this step since direct exposure to EtOH would lead to cell fixation.
  b. Carefully open bone ends using scissors (Figure 1L–1M).
    ***Note:*** Bones should be cut at both ends just enough to expose the marrow for needle insertion, while ensuring that excessive removal of bone material is avoided to prevent reduced cellular yield. If bones were broken during isolation, bone marrow can still be isolated. In this case, cut open the intact bone end and take care during the flushing procedure to avoid flushing the bone from the outside.
  c. Flush out bone marrow directly onto a 100 μm filter placed on top of a 50 mL tube using a 27-gauge needle and a 10 mL syringe containing ice-cold RPMI+2% FBS (Figure 1N).
    ***Note:*** If multiple donors should be pooled, the legs of 5**–**6 mice can be flushed onto the same 100 μm filter. If bone marrow was flushed out completely, the insides of bones will turn white (Figure 1O, top corners).
4. Prepare bone marrow single cell suspensions.
  a. Triturate bone marrow using the plunger of 3 mL syringe as a pestle
  b. Use ice-cold RPMI+2% FBS to rinse filter and pestle with 5 mL of RPMI+2% FBS
  c. Top the volume up to 40 mL.
  d. Centrifuge at 700 × *g* for 5 min at 4°C.
  e. Discard the supernatant.
  f. Dependent on the downstream procedure, either continue with ACK lysis (for bone marrow progenitor analysis via flow cytometry, step 4 f. (i)) or gradient centrifugation (for DC differentiation cultures, step 4 g.).
    i. If continuing with ACK lysis, remove the supernatant completely.
    ii. Resuspend the pellet directly in 500 μL of ACK lysis buffer per mouse.
      ***Note:*** If bone marrow of multiple mice is pooled, scale up the volume of ACK lysis buffer accordingly.
    iii. Incubate for 5 min at 18**–**22°C.
      ***Note:*** If you experience unexpected viability issues resulting from red blood cell lysis, titrate down the incubation time with ACK lysis buffer and/or the amount of lysis buffer. Contrary, if lysis appears to be incomplete, titrate up the incubation time with ACK lysis buffer and/or the amount of lysis buffer.
    iv. Stop lysis by adding 4 mL of RPMI+2% FBS and vortex.
    v. Passively filter the cell suspension via a 40 μm filter into a conical 15 mL tube.
    vi. Rinse tube and subsequently the filter with additional 5 mL of RPMI+2% FBS.
    vii. Centrifuge at 700 × *g* for 5 min at 4°C.
    viii. Discard supernatant, keep the tube upside down, and dip on a paper towel to remove residual buffer.
    ix. Resuspend the pellet in 1 mL of FACS buffer
    x. Top up to 10 mL and take an aliquot for cell counting.
    xi. Continue with cell counting as described in step 5 before returning to step 4 f (xii).
    xii. Centrifuge at 700 × *g* for 5 min at 4°C.
    xiii. Discard supernatant, keep the tube upside down, and dip on a paper towel to remove residual buffer.
    xiv. Resuspend the pellet in FACS buffer at the concentration of 1·10^8^ cells per mL.
    xv. Proceed with the antibody staining as described in step 16.
  g. If continuing with the gradient variant for DC differentiation cultures, passively filter the cell suspension via a 40 μm filter into a conical 15 mL tube.
  h. Rinse the 50 mL tube with another 5 mL RPMI+2% FBS and pour it over the filter once more to collect residual cells.
  i. Centrifuge at 700 × *g* for 5 min at 4°C.
  j. Discard the supernatant by inverting the tube.
  k. Cap the tube and spin again until the centrifuge reaches 700 × *g* and immediately discontinue the run.
  l. Completely remove the left-over supernatant using a pipette.
  m. Resuspend in a minimum of 5 mL of FBS-free RPMI-1640 (18**–**22°C).
  n. Gently layer the cell suspension over an equal volume of Lympholyte-M separation medium (≥5 mL each), while avoiding mixing (Figure 1P left, see Methods Video S1).
    **CRITICAL:** Invert Lympholyte-M three times before usage and aliquot into a desired number of conical tubes depending on the experiment size. Equilibrate Lympholyte-M gradient separation medium and FBS-free RPMI-1640 to 18**–**22°C before use, as lower temperatures alter solution densities. Additionally, bubbles will form from mixing the Lympholyte-M which have to settle before usage. Protect light-sensitive Lympholyte-M from light exposure. Do not exceed the manufacturer’s recommended separation capacity of 20·10^6^ (twenty million) nucleated cells per mL of medium. Ensure sufficient gradient volume so that lymphocytes at the interface can be harvested without contaminating cells from the erythrocyte-rich pellet. We recommend resuspending bone marrow in a minimum of 2 mL FBS-free RPMI per mouse. Nevertheless, a minimum of 5 mL of cell suspension in RPMI-1640 and 5 mL of Lympholyte-M is required to run the gradient effectively. Do not mix cell suspension with Lympholyte-M during the overlay process as lymphocytes cannot be recovered anymore.
    Methods Video S1. Isolation of murine bone marrow leukocytes via gradient centrifugation This video refers to protocol steps 4. n–p.
  o. Spin at 1,000 x *g* for 20 min at 18**–**22°C with the brake turned off or set to minimum.
    **CRITICAL:** Ensure that the centrifuge and all inlays are at 18**–**22°C. The brake must be turned off or set to the lowest level to ensure the interface is not disrupted when the run is over. Following gradient centrifugation at 1,000 × *g*, all subsequent centrifugation steps are again performed with rotor acceleration and brake set to maximum.
  p. Take off the lymphocytes that accumulate at the interface between Lympholyte-M and RPMI-1640 with a 10 mL pipette in one flowing motion (see Methods Video S2).
    Methods Video S2. Medium exchange procedure during bone marrow-based DC differentiation on day 4 This video refers to protocol step 13.
  q. Transfer collected cells to a 15 mL tube.
  r. Top up to 10 mL with ice-cold RPMI-1640 and vortex for 10**–**20 s.
    ***Note:*** When low cell amounts are processed, lymphocytes might not become visible at the interface. Still continue to harvest at the interface. Ensure to include as little Lympholyte-M as possible when collecting cells at the interface. When preparing bone marrow from adult C57BL/6J mice, roughly 30**–**40·10^6^ cells can be expected as yield per mouse prior to gradient centrifugation.
    **CRITICAL:** Vortexing after harvesting the interface and the subsequent dilution with RPMI-1640 is critical to ensure that residual Lympholyte-M does not prohibit pellet formation at the bottom of the tube.
  s. Centrifuge at 700 × *g* for 5 min at 4°C.
  t. Discard the supernatant and resuspend pellets in 10 mL RPMI+2% FBS.
  u. Take an aliquot for cell counting.
5. Cell counting using an improved Neubauer chamber.
  ***Note:*** Following gradient centrifugation roughly 15**–**20·10^6^ cells per mouse are expected.
  a. Take an aliquot of the cell suspension and perform predilution of the aliquot dependent on the number of mice pooled.
    ***Note:*** Write down the pre-dilution factor chosen: *f*_pre-dilution_, it will be needed for cell concentration calculation.
    ***Note:*** There are 9 large squares in the improved Neubauer counting chamber, each with the surface area of 1 mm^2^. We recommend to use the four large corner squares (corner squares are subdivided into 4·4 smaller squares of 0.25·0.25 mm^2^). Ensure that during counting 50**–**100 cells can be found per large quadrant.
  b. Use 10 μL of pre-diluted single cell suspension for counting and transfer it into the improved Neubauer chamber.
    ***Note:*** Only count round, intact, cells showing a brightly glowing rim (known as halo in phase contrast optical microscopy) which are characterized by the classical size of lymphocytes as shown in Figure 2.
    **Figure 2:**
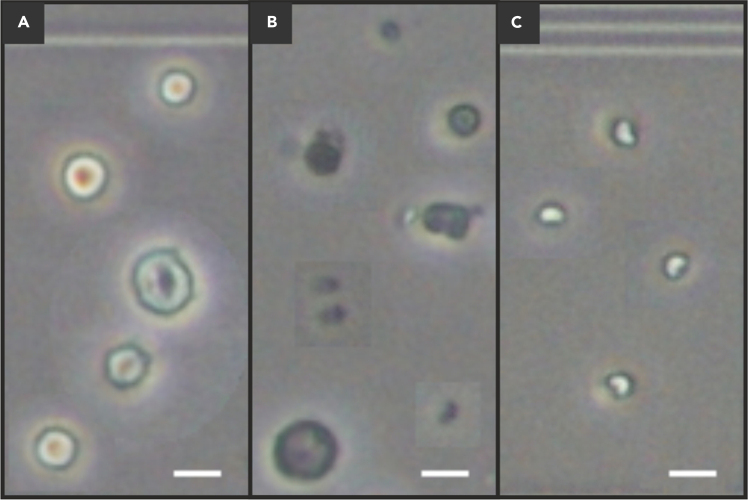
Schematic representation of different cell types and stages during cell counting using an improved Neubauer chamber Owing to the fact that it is statistically impossible to capture exemplary morphologies and cell types within the same field of view, cells and debris were collected from 3 different counting chamber images and pasted together in the corresponding panels. Apart from the copy-paste actions no further image modifications were implemented. Scale bars = 10 μm. (A) Living cells to be counted. (B) Dead cells. (C) Erythrocytes.
  c. Count the number of cells on at least 2 large squares and average: *N*_avg_ = *N*_total cell count_/*N*_large squares counted_
    ***Note:*** The Neubauer chamber constant for a large 1 mm^2^ quadrant, is *k* = 10^4^ mL^-1^. Calculate the concentration of cells utilizing the formula: *c* (cells/mL) = *N*_avg_ · *f*_pre-dilution_ · *k*.
    If the total cell number is desired, multiply the calculated concentration with the volume of the cell suspension. Expected cell counts per donor are displayed in Table 1.
    ***Note:*** Alternatively, dyes such as trypan blue can be used for live-dead cell discrimination on top to phenotypical and morphological features, if the necessary safety measures are taken. Further, cell counts could be determined using automated cell counters, if these are adapted to small cell sizes.

**Table 1:** Expected leukocyte yields of generated single cell suspensions

Bone marrow preparation variant	·10^6^ cells/mouse	·10^6^ cells needed for progenitor analysis	·10^6^ cells needed for differentiation cultures
via Lympholyte-M gradient	∼20	-	20
via ACK lysis	∼35	10	-
  d. Following cell count determination, single cell suspensions can be used in downstream differentiation assays.
  e. Table 1 contains information on expected outcomes and cell numbers required for downstream procedures.
    ***Note:*** If you arrived at the end of step 5 after performing ACK lysis, please return to step 4. f. (xii).
6. Centrifuge at 700 × *g* for 5 min at 4°C.
7. Discard the supernatant.
8. Resuspend pellets in DC differentiation medium to reach a concentration of 2·10^6^ cells per mL.
9. Keep resuspended cells on ice until further use.

***Note:*** Step 9 marks a potential pausing point of up to 3 h. In addition to differentiation of *bona fide* DC subsets from murine bone marrow, a quick buffer exchange allows the direct usage of generated bone marrow single cell suspensions for flow cytometry, cell sorting, functional assays, enrichment procedures, or sequencing techniques.

### Differentiation of *bona fide* DC subsets from murine bone marrow single-cell suspensions

**Timing: 6 days of differentiation with each 30 min of work on day 0, 4, and 6**

This step describes the differentiation of FLT3L-dependent DC populations in the presence or absence of GM-CSF. The protocol utilizes working concentrations of 200ng/mL of FLT3L and 100U/mL of GM-CSF.

10. Supplement the required amount of single cell suspension (in DC differentiation medium) with 200 ng/mL FLT3L.

***Note:*** If required, include control wells without FLT3L.

11. Seed 5 mL of cell suspension (equaling 10·10^6^ cells) per well of a 6-well plate.

***Note:*** To cross-compare differentiation in the presence or absence of GM-CSF, seed at least two individual wells (per biological replica). While one well will receive only FLT3L on day 4, the other well will be additionally supplemented with GM-CSF on day 4.

12. Incubate cells at 37°C in a 5% CO_2_ atmosphere with a relative humidity (rH) of 95% for a total of four days.

***Note:*** This assay format can be adapted to any x-well-plate by adjusting the cell number and total volume in proportion to the well’s growth surface area, while maintaining constant growth factor concentrations. It is ideal to seed two wells for FLT3L differentiation per one well of FLT3L+GM-CSF differentiation to yield similar cell numbers on day 6.

13. Perform medium renewal on day 4. Therefore,
  a. Tilt the plate and carefully remove 50% of media at the well wall without disturbing the cells as shown in Methods Video S2.
    **CRITICAL:** As developing DCs are non-adherent or only loosely adherent in these cultures, improper medium removal may result in significant cell loss. When performing medium renewal, tilt the plate and aspirate the medium slowly using a pipette positioned against the wall of the well, just below the upper surface of the medium and as far away from the cell layer as possible. Add fresh medium slowly against the wall of the well and allow it to run down gently as displayed in Methods Video S2. Do not pipette directly onto the growth surface.
  b. Replace the removed media volume with an equivalent amount of fresh DC differentiation medium supplemented with 200 ng/mL FLT3L ± 200 U/mL GM-CSF (sufficient to reach the final concentration of 100 U/mL GM-CSF per well).
  c. Supplement wells receiving FLT3L alone with an amount of acetic acid equivalent to the volume of GM-CSF added.
14. Continue the incubation for another two days.

***Note:*** Even though we have tested that the final acetic acid concentration of 4 μM in FLT3L cultures is incapable of inducing CD103 and CD301b expression or other phenotypic changes in the marker backbone, we still recommend its inclusion as control.

15. Harvest differentiated cells on day 6.
  a. Carefully rinse the growth surface 4**–**5 times with the medium already contained in the well using a 5 mL pipette.
  b. Transfer the cell suspension to a 15 mL tube.
  c. Rinse well with 1 mL of EDTA-containing sort buffer.
  d. Transfer the suspension to the corresponding 15 mL tube.
  e. Ensure that all cells are harvested using a brightfield or phase contrast microscope to inspect the growth surface.
  f. Top up the 15 mL tube containing the harvested cell suspension to 10 mL with sort buffer.
  g. Determine the cell count as described in step 5.
  h. Cells are ready for use in downstream procedures.

***Note:*** Ensure to perform a buffer exchange in dependence on the downstream application. Table 2 contains anticipated cellular yields after 6 days of differentiation.

**Table 2:** Expected cellular yields retrieved per well (of a 6-well-plate) post 6 days of differentiation *ex vivo*

Culture condition	·10^6^ cells
Culture start	10
FLT3L	∼2.8
FLT3L + GM-CSF (added at d4)	∼4.5

### Antibody staining of total bone marrow single-cell suspensions for flow cytometric analysis of the myeloid progenitor compartment

**Timing: 90 min**

This step describes the labeling of bone marrow single cell suspensions resulting from step 4 f. (xiv) for flow cytometry.

***Note:*** Dependent on the cell numbers required for analysis, this protocol step can be flexibly scaled up or down. For scaling, keep a constant target (cell number) to detection (staining cocktail volume) ratio. For downscaling, do not stain in less than 30 μL of antibody cocktail, even if lower cell numbers are analyzed.

16. Transfer 100 μL of cell suspension (10·10^6^ cells) per well to a 96-well V-bottom plate and keep cells on ice until the antibody cocktail is prepared.

***Note:*** Alternatively, staining of cells can also be performed in FACS tubes or 15 mL tubes.

17. Prepare the primary antibody cocktail by diluting fluorescently labeled antibodies in ice-cold FACS buffer according to Table 3.

**Table 3:** Primary antibody cocktail for flow cytometric analysis of murine bone marrow myeloid progenitor compartment

Label/Fluorophore	Antigen	Clone	Dilution	Company	#Catalog
-	FcγRIV	9E9	1:400	BioLegend	149502
BV650	FcγRIIB/III	93	1:200	BD Biosciences	751690
Biotin	CD3ε	145-2C11	1:200	BioLegend	100304
Biotin	Ly-6G	1A8	1:400	BioLegend	127604
Biotin	Ter-119	TER-119	1:800	BioLegend	116204
Biotin	CD19	6D5	1:400	BioLegend	115504
BV421	CD34	SA376A4	1:200	BioLegend	152208
PE-Cy7	CD115	AFS98	1:200	BioLegend	135524
PE	CD135	A2F10	1:200	eBioscience (Thermo Fisher Scientific)	12-1351-82

***Note:*** A total of 100 μL antibody cocktail is required per well.

***Note:*** We recommend to use a 96-well V-bottom plate to ensure proper sedimentation of the cells by centrifugation and to limit the loss of cells during the staining procedure. Moreover, the protocol presented here is optimized to work with a minimum amount of antibody cocktail volume. If a higher cell number should be stained for analysis, thus requiring the use of FACS tubes or 15 mL tubes, all volumes including cell suspension, antibody cocktails (while maintaining the antibody dilution), and washing buffers must be scaled up accordingly.

18. Centrifuge cells at 700 × *g* for 5 min at 4°C.
19. Discard the supernatant by rapidly inverting the plate, keep the plate up-side down, and gently tap on a paper towel allowing for removal of residual buffer.
20. Resuspend in 100 μL of primary antibody cocktail per well (Table 3).

**CRITICAL:** Check through the bottom of the plate, if any aggregates are present. Continue the resuspension procedure, if necessary, thereby securing homogenous labeling of cells with staining antibodies.

***Note:*** The employed dilutions of antibodies partially depend on the setup of the employed flow cytometer. The antibody dilutions utilized in this protocol are optimized for a five laser-line BD LSR Fortessa SORP equipped with the setup described in Table 4.

21. Incubate samples for 20 min at 4°C in the dark.

**Table 4:** BD LSR Fortessa SORP configuration and channel usage

Laser line	Long pass filter	Band pass filter	Fluorochrome	Antigens (BM DIF) Single step stain	Antigens (BM DIF) Primary/Secondary stain	Antigens (Progenitor analysis)
355 nm	690	740/35	BUV737	CD3ε/CD19	Siglec-H	Siglec-H
	410	470/100	DAPI±BUV496	DAPI only	DAPI+CD3ε +CD19	DAPI+CD3ε+CD19 +Ter119+Ly6G
	-	379/28	BUV395	NK1.1	NK1.1	NK1.1
405 nm	690	710/50	BV711	–	–	–
	630	670/50	BV650	CD16/CD32 (FcγRIIB/III)	CD16/CD32 (FcγRIIB/III)	CD16/CD32 (FcγRIIB/III)
	600	610/20	BV605	–	–	CD45
	545	585/42	BV570	Ly-6C	Ly-6C	Ly-6C
	505	525/50	V500	MHC-II (I-A/I-E)	MHC-II (I-A/I-E)	MHC-II (I-A/I-E)
	-	450/50	BV421	XCR1	XCR1	CD34
488 nm	685	710/50	PerCP-eF710/ PerCP-Cy5.5	CD172a	CD172a	Sca-1
	505	530/30	FITC/AF488	Siglec-H	CD88	CD172a
	-	488/10	FSC/SSC	–	–	–
561 nm	750	780/60	PE-Cy7	CD301b	CD301b	CD115
	635	670/30	PE-Cy5	B220	B220	B220/βTCR/CD49b
	600	610/20	PE-CF594	CD11c	CD11c	CD11c
	-	586/15	PE	–	–	CD135
640 nm	750	780/60	APC-Cy7/ APC-Fire750	CD103	CD103	CD117
	690	730/45	AF700	–	–	CD11b
	-	670/14	APC	CD88	–	–
22. Prepare the secondary antibody cocktail as shown in Table 5 including a fluorescently labeled streptavidin (SA) conjugate.

**Table 5:** Secondary antibody cocktail for flow cytometric analysis of murine bone marrow myeloid progenitor compartment

Label/Fluorophore	Antigen	Clone	Dilution	Company	#Catalog
BUV737	Siglec-H	440c	1:200	BD Biosciences	748293
BUV496	Streptavidin	-	1:50	BD Biosciences	612961
BUV395	NK1.1	PK136	1:400	BD Biosciences	564144
BV605	CD45	30-F11	1:400	BioLegend	103140
BV570	Ly-6C	HK1.4	1:400	BioLegend	128030
V500	MHC-II	M5/114.15.2	1:200	BD Biosciences	562366
PerCP-Cy5.5	Sca-1	E13–161.7	1:200	BioLegend	122524
AF488	CD172a	P84	1:800	BioLegend	144024
PE-Cy5	B220	RA3-6B2	1:800	BioLegend	103210
PE-Cy5	βTCR	H57-597	1:2000	eBioscience (Thermo Fisher Scientific)	15-5961-82
PE-Cy5	CD49b	DX5	1:200	eBioscience (Thermo Fisher Scientific)	15-5971-82
PE-CF594	CD11c	HL3	1:400	BD Bioscience	562454
APC-Fire750	CD117	2B8	1:400	BioLegend	105838
AF700	CD11b	M1/70	1:400	BioLegend	101222
23. Top up wells with 60 μL of FACS buffer.
24. Centrifuge cells at 700 × *g* for 5 min at 4°C.
25. Discard the supernatant by rapidly inverting the plate, keep the plates up-side down, and gently tap on a paper towel allowing for removal of residual buffer.
26. Resuspend in 160 μL of FACS buffer.
27. Centrifuge cells at 700 × *g* for 5 min at 4°C.
28. Discard the supernatant by rapidly inverting the plate, keep the plates up-side down, and gently tap on a paper towel allowing for removal of residual buffer.
29. Resuspend in 100 μL of secondary antibody cocktail per well (Table 5).

**CRITICAL:** Check through the bottom of the plate against the light if any aggregates are present. Continue the resuspension procedure, if necessary, thereby securing homogenous labeling of cells with staining antibodies.

***Note:*** The employed dilutions of antibodies partially depend on the setup of the employed flow cytometer. The antibody dilutions utilized in this protocol are optimized for a five laser-line BD LSR Fortessa SORP equipped with the setup described in Table 4.

30. Incubate samples for 20 min at 4°C in the dark.
31. Top up wells with 60 μL of FACS buffer.
32. Centrifuge cells at 700 × *g* for 5 min at 4°C.
33. Discard the supernatant by rapidly inverting the plate, keep the plates up-side down, and gently tap on a paper towel allowing for removal of residual buffer.
34. Resuspend in 160 μL of FACS buffer.
35. Centrifuge cells at 700 × *g* for 5 min at 4°C.
36. Discard the supernatant by rapidly inverting the plate, keep the plates up-side down, and gently tap on a paper towel allowing for removal of residual buffer.
37. Cells are ready for flow cytometric analysis.
38. Resuspend cells in 50 μL of FACS buffer and keep them on ice until analysis.
39. Add 100 μL of DAPI working solution before acquisition on BD LSR Fortessa SORP.

***Note:*** Step 38 marks a potential pause point. By performing cell fixation, the acquisition of samples might be postponed to the next day. However, we highly recommend to analyze all samples at the same day without fixation. Fixation prior to antibody staining can impact antigen structures leading to altered antibody binding patterns. Fixation after antibody staining can impact properties of the used fluorochromes (e.g., reduced signals for B220 PE-Cy5 and NK1.1 BUV395). Thus, we recommend to revisit employed antibody dilutions in scenarios requiring fixation such as intracellular protein analysis. If fixation or analysis on the same day is not an option, it must be tested whether unfixed cells can be acquired the following day.

### Antibody staining of single-cell suspensions from differentiation cultures for flow cytometric analysis

**Timing: Single-step staining option: 50 min; two-step staining (primary/secondary) option: 90 min**

This step describes the labeling of single cell suspensions resulting from bone marrow differentiation (step 15 h.) for flow cytometry.

***Note:*** Two staining strategies are described. In the single-step approach, lineage exclusion is performed directly in the BUV737 channel, allowing all target populations to be labeled and analyzed within approximately 50 min (Advantage: Speed). In the two-step approach, biotinylated antibodies for combined lineage exclusion are incorporated in the primary incubation, while staining with backbone markers and streptavidin-BUV496 takes place in a second staining step. This frees the BUV737 channel for relocation of Siglec-H thus increasing flexibility in backbone panel design. Moreover, it avoids inclusion of two different states of the same BUV737 tandem dye in the same channel which would be incompatible with spectral flow cytometry (Advantages: Precise compensation and panel flexibility). Dependent on the cell numbers required for analysis, this protocol step can be flexibly scaled up or down. For scaling, keep a constant target (cell number) to detection (staining cocktail volume) ratio. For downscaling, do not stain in less than 30 μL of antibody cocktail, even if lower cell numbers are analyzed.

40. Centrifuge at 700 × *g* for 5 min at 4°C.
41. Discard the supernatant by inverting the tube, keep the tube up-side down, and gently tap on a paper towel allowing for removal of residual buffer.
42. Resuspend cell pellets in FACS buffer to reach a concentration of 1·10^6^ cells per mL.
43. Transfer 100 μL of cell suspension per well to a 96-well V-bottom plate and keep cells on ice until the antibody cocktail is prepared.
44. If more flexibility, precise compensation of the dump channel is desired, proceed with the next stage at point 45. This procedure leads to the two-step (primary/secondary) staining.
If the execution speed and less complexity is favored, prepare the staining mix by diluting fluorescently labeled antibodies in ice-cold FACS buffer as described in the Table 6. and proceed with the single-step antibody staining at the point 59.

**Table 6:** Primary Antibody cocktail for flow cytometric analysis of differentiation cultures

Label/Fluorophore	Antigen	Clone	Dilution	Company	#Catalog
-	FcγRIV	9E9	1:400	BioLegend	149502
Biotin	CD3ε	145-2C11	1:400	BioLegend	100304
Biotin	CD19	6D5	1:400	BioLegend	115504
BV650	FcγRIIB/III	93	1:400	BD Biosciences	751690

***Note:*** In both the single-step and two-step staining workflows, the staining panels are intentionally configured to leave the commonly used PE channel vacant, preserving it for potential downstream profiling requirements or backbone expansion.

45. Prepare the primary antibody cocktail by diluting fluorescently labeled antibodies in ice-cold FACS buffer according to Table 6.
46. Additionally, prepare the secondary antibody cocktail by diluting fluorescently labeled antibodies in ice-cold FACS buffer according to Table 7.

**Table 7:** Secondary antibody cocktail for flow cytometric analysis of differentiation cultures

Label/Fluorophore	Antigen	Clone	Dilution	Company	#Catalog
BUV496	Streptavidin	-	1:50	BD Biosciences	612961
BUV395	NK1.1	PK136	1:400	BD Biosciences	564144
BV570	Ly-6C	HK1.4	1:800	BioLegend	128030
V500	MHC-II (I-A/I-E)	M5/114.15.2	1:200	BD Biosciences	562366
BV421	XCR1	ZET	1:200	BioLegend	148216
PerCP-eF710	CD172a	P84	1:800	eBioscience (Thermo Fisher Scientific)	46-1721-82
BUV737	Siglec-H	440c	1:200	BD Biosciences	748293
PE-Cy7	CD301b	URA-1	1:2000	BioLegend	146808
PE-Cy5	B220	RA3-6B2	1:800	BioLegend	103210
PE-CF594	CD11c	HL3	1:800	BD Biosciences	562454
APC-Cy7	CD103	2E7	1:100	BioLegend	121432
AF488	CD88	20/70	1:100	eBioscience (Thermo Fisher Scientific)	53-0882-82

***Note:*** A total of 50 μL antibody cocktail is required per well in both cases. Additionally, 5,000 Precision Count Beads (BioLegend, #424902, stock concentration ∼1·10^6^ n/mL, exact concentration is LOT specific) can be added to the cocktail per well, if cell count determination prior to staining is not sufficient for quantification.

47. Centrifuge cells at 700 × *g* for 5 min at 4°C.
48. Discard the supernatant by rapidly inverting the plate, keep the plates up-side down, and gently tap on a paper towel allowing for removal of residual buffer.
49. Resuspend in 50 μL of primary antibody cocktail per well.

**CRITICAL:** Check through the bottom of the plate against the light if any aggregates are present. Continue the resuspension procedure, if necessary, thereby securing homogenous labeling of cells with staining antibodies.

50. Incubate samples for 20 min at 4°C in the dark.
51. Top up wells with 100 μL of FACS buffer.
52. Centrifuge cells at 700 × *g* for 5 min at 4°C.
53. Discard the supernatant by rapidly inverting the plate, keep the plates up-side down, and gently tap on a paper towel allowing for removal of residual buffer.
54. Resuspend in 160 μL of FACS buffer.
55. Centrifuge cells at 700 × *g* for 5 min at 4°C.
56. Discard the supernatant by rapidly inverting the plate, keep the plates up-side down, gently tap on a paper towel allowing for removal of residual buffer, and resuspend in 160 µL of FACS buffer.
57. Centrifuge cells at 700 × *g* for 5 min at 4°C.
58. Discard the supernatant by rapidly inverting the plate, keep the plates up-side down, and gently tap on a paper towel allowing for removal of residual buffer.
59. If arriving to this step from the immediately preceding point 58 resuspend cells in 50 μL of secondary antibody cocktail per well, as described in Table 7.

If the choice was to utilize the faster staining procedure, and this step follows directly from point 44 (skipping points 45–58 completely) resuspend cells in 50 μL of single-step antibody cocktail per well, as described in Table 8.

***Note:*** Additionally, 5,000 Precision Count Beads (BioLegend, #424902, stock concentration ∼1·10^6^ n/mL, exact concentration is LOT specific) can be added to the cocktail per well, if cell count determination prior to staining is not sufficient for quantification.

**CRITICAL:** Check through the bottom of the plate, if any aggregates are present. Continue the resuspension procedure, if necessary, thereby securing homogenous labeling of cells with staining antibodies.

60. Incubate samples for 20 min at 4°C in the dark.
61. Top up wells with 100 μL of FACS buffer.
62. Centrifuge cells at 700 × *g* for 5 min at 4°C.
63. Discard the supernatant by rapidly inverting the plate, keep the plates up-side down, and gently tap on a paper towel allowing for removal of residual buffer.
64. Resuspend in 160 μL of FACS buffer.
65. Centrifuge cells at 700 × *g* for 5 min at 4°C.
66. Discard the supernatant by rapidly inverting the plate, keep the plates up-side down, and gently tap on a paper towel allowing for removal of residual buffer.
67. Resuspend in 160 μL of FACS buffer.
68. Centrifuge cells at 700 × *g* for 5 min at 4°C.
69. Discard the supernatant by rapidly inverting the plate, keep the plates up-side down, and gently tap on a paper towel allowing for removal of residual buffer.

***Note:*** The employed dilutions of antibodies partially depend on the setup of the employed flow cytometer. The antibody dilutions utilized in this protocol are optimized for a five laser-line BD LSR Fortessa SORP equipped with the setup described in Table 4.

70. Cells are ready for flow cytometric analysis.
71. Resuspend cells in 50 μL of FACS buffer and keep them on ice until analysis. Add 100 μL of DAPI working solution before acquisition on BD LSR Fortessa SORP.

***Note:*** Step 71 marks a potential pause point. By performing cell fixation, the acquisition of samples might be postponed to the next day. However, we highly recommend to analyze all samples at the same day without fixation. Fixation prior to antibody staining can impact antigen structures leading to altered antibody binding patterns. Fixation after antibody staining can impact properties of the used fluorochromes (e.g., reduced signals for B220 PE-Cy5 and NK1.1 BUV395). Thus, we recommend to revisit employed antibody dilutions in scenarios requiring fixation such as intracellular protein analysis.

**Table 8:** Single-step antibody cocktail for flow cytometric analysis of differentiation cultures

Label/Fluorophore	Antigen	Clone	Dilution	Company	#Catalog
-	FcγRIV	9E9	1:400	BioLegend	149502
BUV737	CD3ε	17A2	1:200	BD Biosciences	612803
BUV737	CD19	1D3	1:400	BD Biosciences	612781
BUV395	NK1.1	PK136	1:400	BD Biosciences	564144
BV650	FcγRIIB/III	93	1:800	BD Biosciences	751690
BV570	Ly-6C	HK1.4	1:800	BioLegend	128030
V500	MHC-II (I-A/I-E)	M5/114.15.2	1:200	BD Biosciences	562366
BV421	XCR1	ZET	1:200	BioLegend	148216
PerCP-eF710	CD172a	P84	1:800	eBioscience (Thermo Fisher Scientific)	46-1721-82
FITC	Siglec-H	551	1:100	BioLegend	129604
PE-Cy7	CD301b	URA-1	1:2000	BioLegend	146808
PE-Cy5	B220	RA3-6B2	1:800	BioLegend	103210
PE-CF594	CD11c	HL3	1:800	BD Biosciences	562454
APC-Cy7	CD103	2E7	1:100	BioLegend	121432
APC	CD88	20/70	1:100	BioLegend	135808

### Antibody staining of single-cell suspensions from differentiation cultures for DC subset sorting

**Timing: 50 min**

This step describes the labeling of single cell suspensions for cell sorting resulting from bone marrow differentiation (step 15 h).

***Note:*** Dependent on the cell numbers required for analysis, this protocol step can be flexibly scaled up or down. For scaling, keep a constant target (cell number) to detection (staining cocktail volume) ratio. For downscaling, do not stain in less than 30 μL of antibody cocktail, even if lower cell numbers are analyzed.

**CRITICAL:** Keep samples sterile, if needed, for downstream use in cell culture. Ensure to use sterile solutions, reagents, and consumables, while working under a sterile work bench using aseptic techniques.

72. Centrifuge at 700 × *g* for 5 min at 4°C.
73. Discard the supernatant by inverting the tube and resuspend cells in 1 mL of ice-cold sort buffer.
74. Top up samples to 10 mL with sort buffer.
75. Keep samples on ice while preparing the antibody cocktail displayed in Table 9.

**Table 9:** Antibody cocktail to label cells for cell sorting

Label/Fluorophore	Antigen	Clone	Dilution	Company	#Catalog
-	FcγRIIB/III	2.4G2	1:400	BioXCell	BE0307
PerCP-eF710	CD172a	P84	1:400	eBioscience (Thermo Fisher Scientific)	46-1721-82
FITC	Siglec-H	551	1:100	BioLegend	129604
PE-Cy7	CD301b	URA-1	1:1000	BioLegend	146808
PE-Cy5	B220	RA3-6B2	1:800	BioLegend	103210
PE-CF594	CD11c	HL3	1:400	BD Biosciences	562454
PE	XCR1	ZET	1:400	BioLegend	148204
APC-eF780	Ly-6C	HK1.4	1:800	eBioscience (Thermo Fisher Scientific)	47-5932-82
APC	CD88	20/70	1:100	BioLegend	135808
76. Centrifuge at 700 × *g* for 5 min at 4°C.
77. Discard the supernatant by inverting the tube.
78. Cap the tube and spin again until the centrifuge reaches 700 × *g* and immediately discontinue the run.
79. Remove residual supernatant with a pipette.
80. Resuspend cells in the antibody cocktail to reach a concentration 2.5·10^6^ cells per 100 μL.

**Table 10:** BD Aria II SORP configuration and channel usage

Laser line	Long pass filter	Band pass filter	Fluorochrome	Antigen
355 nm	440	450/50	DAPI	–
	-	386/23	BUV395	–
405 nm	685	710/40	BV711	–
	550	605/52	BV605	–
	505	550/88	BV510	–
	-	450/50	BV421	–
488 nm	650	710/50	PerCP-eF710	CD172a
	505	525/50	FITC	Siglec-H
	-	488/10	SSC-A	–
561 nm	735	780/60	PE-Cy7	CD301b
	635	670/50	PE-Cy5	B220
	600	610/20	PE-CF594	CD11c
	570	586/15	PE	XCR1
640 nm	735	780/60	APC-eF780	Ly-6C
	685	710/50	AF700	–
	-	670/30	APC	CD88

**CRITICAL:** Check through the bottom of the plate against the light if any aggregates are present. Continue the resuspension procedure, if necessary, thereby securing homogenous labeling of cells with staining antibodies.

***Note:*** The employed dilutions of antibodies partially depend on the setup of the employed flow cytometer. The antibody dilutions utilized in this protocol are optimized for BD Aria II SORP equipped with the setup described in Table 10.

81. Incubate cells for 20 min at 4°C in the dark.
82. Resuspend samples briefly after 10 min to ensure homogenous labeling.
83. Top up with 10 mL of ice-cold sort buffer and vortex.
84. Centrifuge samples at 700 × *g* for 5 min at 4°C.
85. Discard the supernatant by inverting the tube.
86. Cap the tube and spin again until the centrifuge reaches 700 × *g* and immediately discontinue the run.
87. Remove residual supernatant with a pipette.
88. Resuspend cells in 1 mL of ice-cold sort buffer with a pipette.
89. Top up samples to 10 mL, and vortex.
90. Centrifuge samples at 700 × *g* for 5 min at 4°C.
91. Discard the supernatant by inverting the tube.
92. Cap the tube and spin again until the centrifuge reaches 700 × *g* and immediately discontinue the run.
93. Remove residual supernatant with a pipette.
94. Resuspend cells in 200 μL of ice-cold sort buffer.
95. Keep samples on ice until sorting.

### Sample acquisition

**Timing: Flow cytometer setup: 45 min. Sample acquisition: 1 min for differentiation cultures, 2–3 min for progenitor analysis**

**Timing: Sorter setup 1.5 h. Sample acquisition: 5 min for the sort of 1 million differentiated cells yielding 25,000 cells of the scarcest DC subset.**

This step outlines the setup of a BD Aria II SORP for sorting cell suspensions generated in step 95 or a BD LSR Fortessa SORP for analyzing suspensions from step 38 or step 71. Additional procedures required specifically for the BD Aria II will be highlighted below. The final section details data acquisition.

96. Start up the BD Aria II SORP sorter.
  a. Fill up sheath fluid and EtOH tanks, if necessary, empty the waste container, and close tank lids.
  b. Detach the pressured air line from the EtOH tank and de-pressurize the EtOH tank, if necessary.
  c. Detach the sheath line from the EtOH tank and attach it to the sheath tank.
  d. Attach the pressured air line to the sheath tank.
  e. Apply air pressure to the fluidics systems according to your site-specific conditions.
  f. Connect the cooling loop lines to the respective target tube holder.
  g. Start the BD Aria II sorter.
  h. Start the thermostat (set to 4°C).
  i. Start the computer, and DIVA software.
  j. Site specific: Turn on manually the lasers that do not start up automatically using the “Coherent Connection” software of BD (here used to start up the UV laser).
  k. Perform fluidics start-up by following the instructions within DIVA software.
  l. Following start-up, remove the closed loop nozzle and replace it with an 85 μm nozzle.
  m. Choose the correct cytometer setup in DIVA software fitting the installed nozzle and filter set-up.
  n. Switch on the stream, adjust its settings.
  o. Let the machine run for 20 min allowing for the stabilization of the stream.
  p. Set the correct stream parameters as target values for the sweet spot and enable stream sweet spot.
  q. Run daily performance check using CS&T beads (BD, CAT#655050) according to the manufacturer’s instructions.
  r. Run Accudrop beads (BD, CAT#345249) according to the manufacturer’s instructions to calibrate droplet formation.
  s. Perform a test sort into target tubes to adjust plate deflection.
  t. Subsequently, the sorter is ready for performing experiment set-up analogously to the BD LSR Fortessa SORP (continuing in step 98).
97. Start up the BD LSR Fortessa SORP flow cytometer.
  a. Check fill levels of sheath fluid and waste tanks.
  b. Turn on the fluidics cart.
  c. Turn on the flow cytometer.
  d. Turn on the computer, and start DIVA software.
  e. Let the flow cytometer run for 15 min allowing for the stabilization and rinsing of the fluidics.
  f. Run daily performance check using CS&T beads according to the manufacturer’s instructions.
  g. Subsequently, the BD LSR Fortessa SORP is ready for performing experiment set-up analogously to the BD Aria II SORP system.
98. Set up an experiment.
  a. Start by creating an experiment.
  b. Create compensation samples including the “unstained control tube”.
  c. Use a fully stained sample to ensure all signals are on scale.
  d. Adjust PMT voltages, if necessary, in the unstained control sample displaying all detection channels in parallel.
  e. Run automatic compensation using single-stained compensation beads as controls (UltraComp eBeads Plus Compensation Beads, eBioscience) labeled with the same lot of antibodies used in the panel.
    **CRITICAL:** Use the same antibody vials applied in the staining procedure to ensure accurate compensation. This is especially important for tandem dyes, which may deteriorate over time altering their spectral characteristics.
  f. As a general guideline, pre-dilute antibodies conjugated to PE or PE-based tandem dyes 1:4,000 in FACS buffer, and dilute all other antibodies 1:2,000 in FACS buffer.
  g. Add one drop of thoroughly vortexed UltraComp eBeads Plus Compensation Beads to 400 μL FACS buffer and vortex again.
    ***Note:*** Adjust both the bead volume and number of drops according to the total number of compensations needed.
    **CRITICAL:** During compensation, signals of the positive population in the compensation sample should be around 10^4^ at a BD LSR Fortessa SORP and BD Aria II SORP, but higher than in measured samples and clearly separated from the negative bead population to improve compensation accuracy.
    ***Note:*** We recommend using compensation beads to generate single-stained controls. If beads are unavailable, or if certain dyes cannot be compensated using beads (e.g., fruit-derived fluorescent proteins, proliferation dyes, or fixable viability dyes), single-stained controls may be prepared from cell suspensions instead. When using cells for compensation, ensure that the staining produces signal intensities that clearly separate positive and negative populations (base-line separated), both of which should exhibit the same level of autofluorescence. If a marker is compensated that is able to stain all cells (or all cells with a similar autofluorescence), spike in unstained cells after labeling. Alternatively, amine-reactive or fruit-color compensation beads can be used for compensation of fixable viability dyes and fruit color proteins. If UltraComp eBeads Compensation Beads from Invitrogen are used for performing compensation as described here, beads can be used after staining for up to 3 days if the antibody-coated beads are stored in a fixative according to the manufacturer. If the antibody-coated beads are stored in FACS buffer, they are stable for 1 week. It is highly recommended to treat the antibody-coated beads identically to cell samples as procedures such as fixation might alter the spectral properties of employed dyes (particularly important for spectral flow cytometers).
  h. Combine 100 μL of the pre-diluted compensation beads with 100 μL of the pre-diluted antibodies in FACS tubes.
  i. Mix by vortexing and incubate for 10 min at 18**–**22°C in the dark.
  j. Acquire the compensation samples immediately after labeling.
  k. Remove the “unstained control tube”, calculate the compensation matrix, and press “Link and save”.
    ***Note:*** For comprehensive guidance on general flow cytometer/sorter setup and operation, consult Cossarizza et al., 2021, which provides recommendations for the use of flow cytometry and cell sorting in immunological research.^22^ Additionally, machine and software specific guidelines are provided by the flow cytometer manufacturers, e.g. the “BD FACSDiva Software Quick Reference Guide for the BD LSR II or BD LSRFortessa”.
  l. After completing instrument setup, acquire samples on a BD LSRFortessa SORP, sort them using a BD Aria II SORP, or use any equivalent flow cytometer or sorter.
    i. For sorting: Filter samples directly before sorting via a pre-wetted 35 μm filter (Corning Falcon®, CAT#352235).
    ii. Choose 4-way sort and 4-Way Purity as purity setting.
    iii. Sort into low-binding tubes (Corning Falcon®, CAT#352063) containing 400 μL of ice-cold DC differentiation medium.
      ***Note:*** For regular flow cytometry, samples originating from either bone marrow differentiation cultures or directly from the preparation of primary bone marrow cells usually do not require filtration prior to analysis.
    iv. After sorting, perform reanalysis of a fraction of the sample to ensure sort quality.
  m. Process data using appropriate analysis software.
    ***Note:*** In this study, FlowJo (BD Biosciences) was used for data evaluation.
  n. After sorting or flow cytometric analysis, perform cleaning and shutdown procedures in accordance with the manufacturer’s device-tailored routines and your local protocols.
    ***Note:*** For DC subset analysis post differentiation at a flow cytometer, we recommend to acquire 20,000 cells to have sufficient events for every DC subset of interest. For analysis of myeloid progenitors from bone marrow, we recommend to acquire 2·10^6^ cells.

## Expected outcomes

**Figure 3:**
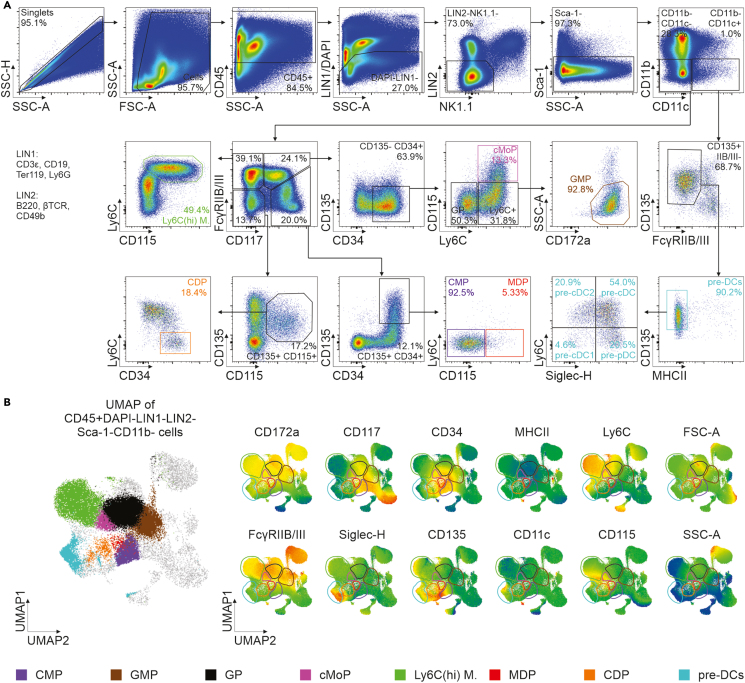
Identification of myeloid progenitors in murine bone marrow (A) Annotation of myeloid progenitors in bone marrow single cell suspensions of C57BL/6 J mice via flow cytometry. The provided annotation approach is based on the combined work of Liu et al.^23^ and Schlitzer et al.^24^ (B) Dimensionality reduction via Uniform Manifold Approximation and Projection (UMAP) of 30,000 cells from CD45+DAPI-LIN- cells. Gates in (A) were mapped onto the UMAP and color coded (left) while visualizing the expression of population defining markers as expression heat maps (right). Unbiased analysis was performed using the FlowJo plugins downsample and UMAP.^25^ LIN=lineage; UMAP=uniform manifold approximation and projection; CMP=common myeloid progenitor; GMP=granulocyte-macrophage progenitor; GP=granulocyte progenitor; cMoP=common monocyte progenitor; M.=monocytes; MDP=monocyte-dendritic cell progenitor; CDP=common dendritic cell progenitor.

**Figure 4:**
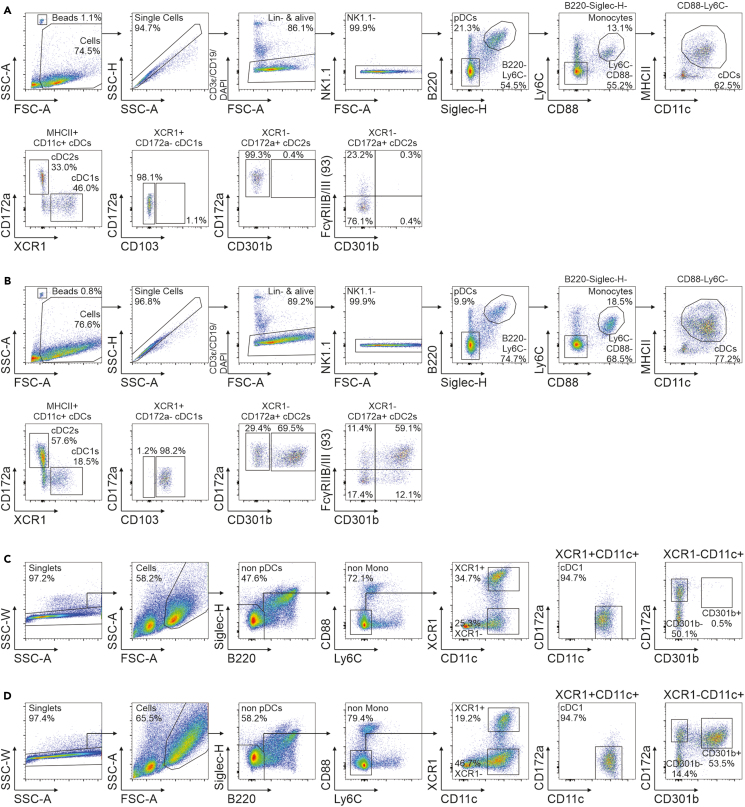
Annotation of cDC subsets during flow cytometric analysis and cell sorting (A and B) Gating of cDC subpopulations resulting from (A) FLT3L (d0) and (B) FLT3L (d0) +GM-CSF (d4) cultures after a total of 6 days of *ex vivo* differentiation. (C and D) Sort strategy of cDC subpopulations resulting from (C) FLT3L (d0) and (D) FLT3L (d0) +GM-CSF (d4) cultures after a total of 6 days of *ex vivo* differentiation.

After data acquisition, samples are evaluated utilizing FlowJo Software (BD). In flow cytometry data, populations of interest are identified via manual gating according to their phenotypic characteristics displayed in Figures 3 and 4. The annotation of myeloid progenitor identification was developed in accordance with Liu et al. and Schlitzer et al.^23^^,^^24^ The gating of DC subsets resulting from *ex vivo* differentiation was developed and validated in Amon et al.^1^

**Figure 5:**
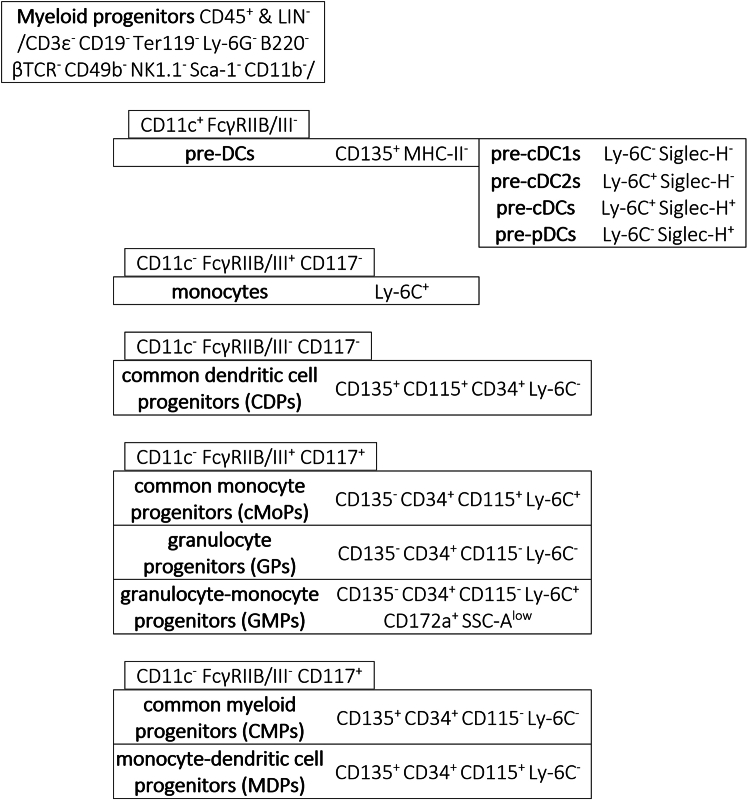
Detailed annotation of the myeloid progenitor populations

Myeloid progenitors in murine bone marrow are annotated within the single, live, CD45^+^, and LIN^-^ (CD3ε, CD19, Ter119, Ly-6G, B220, βTCR, CD49b, NK1.1, Sca-1, CD11b) cell pool (Figures 3A and 5). Within CD11c^+^ cells, pre-DCs are CD135^+^FcγRIIB/III^-^MHC-II^-^ and separated into pre-cDC1s (Ly-6C^-^Siglec-H^-^), pre-cDC2s (Ly-6C^+^Siglec-H^-^), pre-cDCs (Ly-6C^+^Siglec-H^+^), and a contaminating population of lymphoid pre-pDCs^26^ (Ly-6C^-^Siglec-H^+^). Within CD11c^-^ cells, Ly-6C^+^ monocytes (FcγRIIB/III^+^CD117^-^Ly-6C^+^CD115^+^), common monocyte progenitors (cMoPs, FcγRIIB/III^+^CD117^+^CD135^-^CD34^+^CD115^+^Ly-6C^+^), granulocyte progenitors (GPs, FcγRIIB/III^+^CD117^+^CD135^-^CD34^+^CD115^-^Ly-6C^-^), granulocyte-monocyte progenitors (GMPs, FcγRIIB/III^+^CD117^+^CD135^-^CD34^+^CD115^-^Ly-6C^+^CD172a^+^SSC-A^low^), common dendritic cell progenitors (CDPs, FcγRIIB/III^-^CD117^-^CD135^+^CD34^+^CD115^+^Ly-6C^-^), common myeloid progenitors (CMPs, FcγRIIB/III^-^CD117^+^CD135^+^CD34^+^CD115^-^Ly-6C^-^), and monocyte-dendritic cell progenitors (MDPs, FcγRIIB/III^-^CD117^+^CD135^+^CD34^+^CD115^+^Ly-6C^-^) are annotated (Table 11).

**Table 11:** Expected bone marrow progenitor cell counts

Bone marrow progenitor populations	·10^3^ cells/mouse
Ly6C^+^ monocytes	∼320
CMP (common myeloid progenitor)	∼39
MDP (monocyte-dendritic cell progenitor)	∼2.2
CDP (common dendritic cell progenitor)	∼7.4
cMoP (common monocyte progenitor)	∼34
GMP (granulocyte-monocyte progenitor)	∼77
GP (granulocyte progenitor)	∼130
Siglec-H^-^ Ly6C^+^ pre-DCs	∼6.7
Siglec-H^+^ Ly6C^+^ pre-DCs	∼18
Siglec-H^+^ Ly6C^-^ pre-DCs	∼6
Siglec-H^-^ Ly6C^-^ pre-DCs	∼1.2

To validate the separation of individual populations, while highlighting the similarities between related progenitors, we performed unbiased clustering and dimensionality reduction via Uniform Manifold Approximation and Projection (UMAP). In general, UMAP is a computational method that reduces the number of dimensions inside complex datasets by embedding them into a lower-dimensional space while maintaining meaningful relationships between data points. This allows for a global view on phenotypically distinct cell populations by overcoming the constraints of manual gating strategies, which are relying on sequential two-dimensional parameter assessment. In contrast, UMAP captures high-dimensional relationships across all measured markers simultaneously. For that purpose, we installed the UMAP plugin readily available at FlowJo exchange. Detailed step-by-step instructions can be found on https://docs.flowjo.com/flowjo/plugins-2/plugin-demonstration-videos/. UMAP was performed on the single, live, CD45^+^LIN^-^ (CD3ε, CD19, Ter119, Ly-6G, B220, βTCR, CD49b, NK1.1, Sca-1, CD11b) cell pool using FSC-A, SSC-A and all markers not utilized for lineage exclusion as parameters for UMAP calculation. Visualization of cluster defining markers and annotation of manually gated populations within the UMAP demonstrated the phenotypic similarity of defined progenitor populations during myeloid differentiation cascades (Figure 3B). By including CX3CR1 BV711 (BioLegend, Clone SA011F11, CAT #149031) and CD45RB AF647 (BioLegend, Clone C363-16 A, CAT #103312) into the currently free BV711 and AF647 channels, the presented panel is suitable for analysis of R1, R2, and R3 monocytes as defined by Menezes et al. and for annotation of pro-DC3, pre-DC2, and DC3 in the bone marrow according to Liu et al. as shown in Amon et al.^1^^,^^27^^,^^28^

Within bone marrow cultures after 6 days of differentiation, B220^+^Siglec-H^+^ plasmacytoid DCs (pDCs), B220^-^Siglec-H^-^CD88^+^Ly6C^+^ monocytes, and B220^-^Siglec-H^-^CD88^-^Ly6C^-^CD11c^+^MHC-II^+^ cDCs can be annotated within the pool of single, living, lineage negative (CD3ε, CD19, NK1.1) cells during flow cytometric analysis (Figure 4A/B). cDCs can subsequently be separated into XCR1^+^CD172a^-^ cDC1s and XCR1^-^CD172a^+^ cDC2s. While these populations are present in cultures containing FLT3L alone or in combination with GM-CSF, the addition of GM-CSF at day 4 post culture start switches the phenotype of all cDC1s from CD103^-^ to CD103^+^. Moreover, GM-CSF facilitates the generation of CD301b^+^ cells within cDC2s. Further, FLT3L cultures intrinsically generate FcγRIIB/III^+^ cDC2s, and supplementing with GM-CSF further increases their proportion within the total cDC2 population. Quantification of cell yields across differentiation conditions can be performed using Precision Count Beads™ (BioLegend) or equivalent (Table 12), following the manufacturer’s instructions.

**Table 12:** Expected counts of dendritic cell subsets after 6 days of differentiation

Dendritic cell subset	Condition	·10^3^ cells/well
cDC1	FLT3L	∼330
CD301b^+^ cDC2	FLT3L	∼1.3
CD301b^-^ cDC2	FLT3L	∼120
cDC1	FLT3L + GM-CSF	∼380
CD301b^+^ cDC2	FLT3L + GM-CSF	∼490
CD301b^-^ cDC2	FLT3L + GM-CSF	∼140

During cell sorting (Figures 4C–4D), individual subsets were annotated analogously to regular flow cytometric analysis except for MHC-II. The anti-MHC-II antibody clone M5/114.15.2 used to detect MHC-II molecules coded in the I-A and I-E locus usually blocks the interaction with cognate T cells. Thus, MHC-II cannot be used for sorting prior to performing T cell:DC co-cultures. cDC subsets generated or sorted from differentiation cultures are suitable for usage in a variety of downstream applications including but not limited to bulk-RNA and ATAC sequencing, metabolomic studies, T cell priming assays, cell transfers, stimulations, cytokine profiling via cytometric bead arrays, phagocytosis assays, or migration studies. Detailed protocols for downstream functional assessment of generated cDC populations are provided in the Guidelines for mouse and human DC functional assays by Clausen et al.^2^ While the presented panel for the analysis of differentiated DCs contains the most important markers for their identification, we recommend to use optimized panels for the detection of DCs, monocytes, or macrophages directly isolated from a variety of tissues as developed previously.^3^^,^^21^^,^^29^^,^^30^

## Limitations

In its current configuration, the panel for BD LSR Fortessa SORP analysis of DC subsets post bone marrow differentiation allows screening of additional markers only in the PE channel, representing the sole available slot for non-tandem dyes in the fast-track version of the protocol. However, using the longer (two-step) version based on biotinylated anti-lineage antibodies, allows to free up the AF647/APC or FITC/AF488 channel in addition, permitting flexible reassignment of antibodies. This enables the most commonly used non-tandem dye channels to be readily freed for other markers of interest.

The antibody dilutions provided were optimized for a BD LSR Fortessa SORP with five laser lines (355, 405, 488, 561, and 640 nm) and a BD Aria II SORP cell sorter. These dilutions may require adjustment when using different flow cytometers and cell sorters.

In this panel, DAPI is used to discriminate live and dead cells, making the current protocol incompatible with fixation. Replacing DAPI with a fixable viability dye allows for simultaneous fixation and dead cell exclusion. For example, the Zombie UV™ Fixable Viability Kit (BioLegend, CAT#423107) is a suitable alternative, as it is typically detected in the same channel as DAPI on most flow cytometers. This allows to combine our differentiation platform and flow cytometric analysis with the analysis of cytoplasmic/endosomal and nuclear proteins as suggested previously.^1^^,^^21^

To allow profiling of proteins of interest, panels for flow cytometric analysis either have a free channel in AF647/APC or in PE. Additionally, these slots can be switched to either FITC/AF488, PE, or AF647/APC allowing profiling in the most popular non-tandem dye formats.

In its current form, it remains unclear to what extent this protocol can be directly translated to human cell culture systems. Although several key aspects of dendritic cell (DC) biology are conserved between mice and humans (e.g., FLT3L dependency and CD103 expression on cDC1s), other features differ substantially.^31^ For example, FLT3L deficiency affects monocyte development in humans but not in mice. Therefore, the separation of DCs and monocytes in human FLT3L-based culture systems requires careful re-evaluation.^32^ Additionally, human CLEC10A has two orthologs in mice, CD301a (MGL1) and CD301b (MGL2). While human CLEC10A and murine CD301b share ligand specificity for N-acetylgalactosamine (GalNAc), all human cDC2s express CLEC10A.^33^^,^^34^ Consequently, in contrast to the murine system, CLEC10A cannot be used as a marker for a cytokine-induced state in human cDC2s.^1^ Although GM-CSF-based culture systems for the generation of human DCs and DC-like cells have a long-standing history, it remains unclear how timed addition of GM-CSF to FLT3L-based cultures would influence human DC differentiation *ex vivo*.^10^^,^^35^ A comprehensive overview of recent mouse and human DC culture systems, including various cell sources and cytokine combinations used for differentiation, is provided in Lutz et al.^9^

## Troubleshooting

### Problem 1

Low cellular yields and/or viability following bone marrow preparation and gradient centrifugation.

### Potential solution

Multiple reasons can result in lower cellular yields.

• Avoid bone ruptures prior to EtOH dipping resulting in bone marrow cell fixation and thus death (step 2.).
• Avoid cutting off too much bone material prior to flushing out bone marrow (step 3. b.).
• Ensure to equilibrate RPMI without additions (important to maintain the regular RPMI density) and Lympholyte-M, centrifuges as well as centrifuge inlays to 18**–**22°C prior to running gradient centrifugation (step 4. g.-p.).
• Ensure that during overlaying of Lympholyte-M with cell suspensions in RPMI the two liquids are not mixed (step 4. n.).
• Ensure that Lympholyte-M is mixed well but bubble free prior to usage (step 4. n.).
• Ensure to turn off the brake or to set it to a very low setting (step 4. o.).
• Ensure to mix the single cell suspensions well after harvesting the interface post gradient centrifugation (step 4. q.).
• Use mice of at least 8 weeks of age to ensure that bone marrow derived DC-poesis is fully developed.^36^ For consistency, we recommend to use mice no older than 6 months (25 weeks) of age, since multiple factors (e.g., changes in hormonal levels and fertility but also in the stem cell compartment) could alter the output of bone marrow progenitors and differentiated DC subsets.^37^ In any case, we highly recommend to use age- and sex-matched littermate controls, when assessing effects between two or more different conditions.

### Problem 2

Following differentiation, the percentage or yields of DC subsets differs from the expected range (see “expected outcomes”).

### Potential solution

• Ensure to check if growth factors have a similar activity between different vendors and/or LOTs (if possible, in U/mL).
• Perform a titration to determine ideal doses for generating the different DC subpopulations.
• Check if activity measures between different vendors are based on the same assay readout and try to use similar doses (preferentially in U/mL).
• This protocol specifically uses female C57BL6/J mice from Charles River between 8 and 15 weeks of age. Genetic and environmental variables such as the genetic background of the used mouse strain or microbiome likely alter cellular output.^38^ Additionally, age and sex of mice add another variable to the system potentially altering frequencies of progenitors within bone marrow or of DCs following *ex vivo* differentiation.

### Problem 3

Bone marrow single cell suspensions in differentiation assays are depleted/reduced in granulocytes.

### Potential solution

• If this protocol is used to differentiate other immune cells from bone marrow, replace gradient centrifugation with simple ACK or other osmotic lysis procedures (step 4.).
• Gradient centrifugation not only reduces the number of erythrocytes, but also granulocytes which will locate between the erythrocyte pellet and the lymphocyte containing phase within the Lympholyte-M cell separation medium (step 4. g.-q.).

### Problem 4

The resolution of employed markers is insufficient for clear separation of cell populations (see “expected outcomes”).

### Potential solution

Multiple reasons can result in this issue.

• Confirm that the flow cytometer’s laser lines and filters are compatible with the dyes used (see “before you begin”).
• Ensure that PMT voltages are properly set during sample acquisition (steps 96., 97., and 98.).
• Verify that the correct antibody clones were used for staining and re-titrate antibodies according to your current flow cytometer configuration.
• Ensure that the ratio of cell number to detection antibody volume remains constant, which is in particular important for staining of CD135 and CD115 (steps 20., 29., 49., 59., and 80.). If different cell numbers are subject of analysis, scale staining mix volumes up or down accordingly. Staining in V-bottom plates should however not be carried out in volumes lower than 30 μL.
• Keep samples cold at all times. For example, CD115 signal will be lost if samples are kept at temperatures above 4°C.
• Ensure to take the suggested clone of the CD34 detection antibody, since other commercially available clones recognize a CD34 variant exclusively expressed by cell culture cell lines (step 17.).
• If no clear separation between lineage positive and negative cells is seen in the dump channel, re-titrate biotinylated antibodies followed by titration of the fluorescently labeled streptavidin:dye conjugate (see “expected outcomes”). We recommend to keep anti-NK1.1 and anti-Sca-1 antibodies in individual colors. These antibodies are often characterized by a low resolution between negative and positive populations. Further, the anti-NK1.1 antibody has a mouse IgG2a,κ backbone, thereby causing it to be bound by FcγRIV^+^ cells. Thus, not only blockade of FcγRIV using purified clone 9E9 is important, but also individual detection of NK1.1 to avoid the exclusion of FcγRIV^+^ cells such as Ly6C^low^ monocytes in case they are falsely positive for NK1.1.^39^
• Check if the target antigen is expressed in the mouse strain under analysis; this panel was optimized for C57BL/6 J mice. For MHC-II, confirm that the relevant alleles (e.g., I-A^b^, I-A^d^, I-A^q^, I-E^d^, and I-E^b^ for C57BL/6 J mice) are expressed. Moreover, NK1.1 is not expressed by all mouse strains.
• For detailed guidance, see Cossarizza et al., 2021.^22^

### Problem 5

It is unclear how to set the gate for positive and negative populations.

### Potential solution

• Fluorescence minus one (FMO) and/or isotype controls should be employed for individual markers.
• Data can also be visualized using alternative representations, such as density plots, to highlight the center of populations.
• Back-gating routines are recommended to ensure that populations of interest are not prematurely excluded.
• Single-stained controls for the combined dump channel should be used to improve resolution between positive and negative populations, as demonstrated for DCs previously.^30^

## Resource availability

### Lead contact

Further information and requests for resources and reagents should be directed to and will be fulfilled by the lead contact, Diana Dudziak (diana.dudziak@med.uni-jena.de).

### Technical contact

Questions about the technical specifics of performing the protocol should be directed to and will be answered by the technical contacts, Damir Vurnek (damir.vurnek@med.uni-jena.de) and Lukas Amon (lukas.amon@irc.vib-ugent.be).

### Materials availability

This study did not generate new unique reagents.

### Data and code availability

The datasets supporting the current protocol are available from the lead contact upon request.

## Acknowledgments

Parts of this manuscript are included in the PhD thesis of L.A. Schematic illustrations in the graphical abstract are provided by and adapted from Servier Medical Art (https://smart.servier.com) and are licensed under CC BY 4.0 (https://creativecommons.org/licenses/by/4.0/).

We are thankful for the support of Markus Mroz and Uwe Appelt from the Core Unit Cell Sorting and Immunomonitoring of the Friedrich-Alexander-Universität Erlangen-Nürnberg and of Katrin Hornung and Dr. Sabine Baumgart from the Core Facility Cytometry of the Institute of Immunology at Jena University Hospital for consulting on the FACS panel design. We further thank Gert Van Isterdael and the VIB Flow Core Ghent for their valuable support.

This study was funded by several grants, including 10.13039/501100001659German Research Foundation (10.13039/501100001659DFG) and Agence Nationale de la Recherche (ANR) grant DU548/6-1 (431402787), 10.13039/501100001659German Research Foundation (10.13039/501100001659DFG) program TRR305 project B05 (429280966), 10.13039/501100001659German Research Foundation (10.13039/501100001659DFG) program TRR374 project B7 (509149993), DU548/9-1 (515982377), and funding from the Bavarian State Ministry of Science and Art Bayresq.Net “Project IRIS” to D.D. and 10.13039/501100001659German Research Foundation (10.13039/501100001659DFG) program RTG2504 (401821119) to D.D. and C.H.K.L. Further, D.D. is principal investigator and N.R.T. is a postdoctoral fellow funded by the DFG under Germany’s Excellence Strategy – EXC 2051 – Project-ID 390713860. T.V. was supported by the project “Podizanje znanstvene izvrsnosti centra za napredne laserske tehnike (CALTboost)” financed by the European Union – NextGenerationEU through the National Recovery and Resilience Plan 2021–2026 (NRPP). B.N.L. was funded by the European Union. Views and opinions expressed are however those of the authors only and do not necessarily reflect those of the European Union or the European Research Council Executive Agency. Neither the European Union nor the granting authority can be held responsible for them. B.N.L. is supported by ERC grants ASTHMACRYSTALCLEAR (789384) and CRYSTAL-SHINE (101189517), Excellence of Science (EOS) research grants EOS 30565447 and 3G0H1222, iBOF-10.13039/501100003130FWO grant 01GA2817, and 10.13039/501100007229BOF Methusalem grant 01M01521.

## Author contributions

Conceptualization, D.V., B.N.L., D.D., and L.A.; methodology, D.V., L.A., C.H.K.L., and D.D.; experimentation, D.V., L.A., C.H.K.L., G.T., and A.S.; evaluation and visualization, D.V., L.A., I.H., and L.H.; writing – original draft, D.V. and L.A.; writing – review and editing, D.V., G.T., A.S., T.V., I.H., L.T., L.H., C.H.K.L., B.N.L., D.D., and L.A.; supervision, L.A., B.N.L., and D.D.; funding acquisition, T.V., B.N.L., C.H.K.L., and D.D.

## Declaration of interests

The authors declare no competing interests.

## References

[1] Amon L., Vurnek D., Seichter A., Tchitashvili G., Kaszubowski T., Mroz M., Debeuf N., Vogler T., Küpper N., Rengarajan K.R. (2025). GM-CSF and specific type 2 cytokines induce CD103+ and CD301b+ cell states in cDC1s and cDC2s. Cell Rep..

[2] Clausen B.E., Amon L., Backer R.A., Berod L., Bopp T., Brand A., Burgdorf S., Chen L., Da M., Distler U. (2023). Guidelines for mouse and human DC functional assays. Eur. J. Immunol..

[3] Amon L., Seichter A., Vurnek D., Tchitashvili G., Heß I., Heger L., Lehmann C.H.K., Dudziak D. (2024). Protocol for mapping the heterogeneous dendritic cell network across the murine tissue landscape via high-dimensional flow cytometry. STAR Protoc..

[4] Kim T.-G., Kim S.H., Park J., Choi W., Sohn M., Na H.Y., Lee M., Lee J.W., Kim S.M., Kim D.-Y. (2018). Skin-Specific CD301b+ Dermal Dendritic Cells Drive IL-17−Mediated Psoriasis-Like Immune Response in Mice. J. Invest. Dermatol..

[5] Mayer C.T., Ghorbani P., Nandan A., Dudek M., Arnold-Schrauf C., Hesse C., Berod L., Stüve P., Puttur F., Merad M., Sparwasser T. (2014). Selective and efficient generation of functional Batf3-dependent CD103+ dendritic cells from mouse bone marrow. Blood.

[6] Sathe P., Pooley J., Vremec D., Mintern J., Jin J.-O., Wu L., Kwak J.-Y., Villadangos J.A., Shortman K. (2011). The Acquisition of Antigen Cross-Presentation Function by Newly Formed Dendritic Cells. J. Immunol..

[7] Helft J., Böttcher J., Chakravarty P., Zelenay S., Huotari J., Schraml B.U., Goubau D., Reis e Sousa C. (2015). GM-CSF Mouse Bone Marrow Cultures Comprise a Heterogeneous Population of CD11c+MHCII+ Macrophages and Dendritic Cells. Immunity.

[8] Inaba K., Inaba M., Romani N., Aya H., Deguchi M., Ikehara S., Muramatsu S., Steinman R.M. (1992). Generation of large numbers of dendritic cells from mouse bone marrow cultures supplemented with granulocyte/macrophage colony-stimulating factor. J. Exp. Med..

[9] Lutz M.B., Ali S., Audiger C., Autenrieth S.E., Berod L., Bigley V., Cyran L., Dalod M., Dörrie J., Dudziak D. (2023). Guidelines for mouse and human DC generation. Eur. J. Immunol..

[10] Lutz M.B., Kukutsch N., Ogilvie A.L., Rössner S., Koch F., Romani N., Schuler G. (1999). An advanced culture method for generating large quantities of highly pure dendritic cells from mouse bone marrow. J. Immunol. Methods.

[11] Kirkling M.E., Cytlak U., Lau C.M., Lewis K.L., Resteu A., Khodadadi-Jamayran A., Siebel C.W., Salmon H., Merad M., Tsirigos A. (2018). Notch Signaling Facilitates In Vitro Generation of Cross-Presenting Classical Dendritic Cells. Cell Rep..

[12] Ou F., Ferris S.T., Kim S., Wu R., Anderson D.A., Liu T.-T., Jo S., Chen M.Y., Gillanders W.E., Murphy T.L., Murphy K.M. (2023). Enhanced in vitro type 1 conventional dendritic cell generation via the recruitment of hematopoietic stem cells and early progenitors by Kit ligand. Eur. J. Immunol..

[13] Hieronymus T., Gust T.C., Kirsch R.D., Jorgas T., Blendinger G., Goncharenko M., Supplitt K., Rose-John S., Müller A.M., Zenke M. (2005). Progressive and Controlled Development of Mouse Dendritic Cells from Flt3+CD11b+ Progenitors In Vitro1. J. Immunol..

[14] Naik S.H., Proietto A.I., Wilson N.S., Dakic A., Schnorrer P., Fuchsberger M., Lahoud M.H., O’Keeffe M., Shao Q.x., Chen W.f. (2005). Cutting Edge: Generation of Splenic CD8+ and CD8− Dendritic Cell Equivalents in Fms-Like Tyrosine Kinase 3 Ligand Bone Marrow Cultures1. J. Immunol..

[15] Edelson B.T., KC W., Juang R., Kohyama M., Benoit L.A., Klekotka P.A., Moon C., Albring J.C., Ise W., Michael D.G. (2010). Peripheral CD103+ dendritic cells form a unified subset developmentally related to CD8α+ conventional dendritic cells. J. Exp. Med..

[16] Kumamoto Y., Linehan M., Weinstein J.S., Laidlaw B.J., Craft J.E., Iwasaki A. (2013). CD301b+ Dermal Dendritic Cells Drive T Helper 2 Cell-Mediated Immunity. Immunity.

[17] Bogunovic M., Ginhoux F., Helft J., Shang L., Hashimoto D., Greter M., Liu K., Jakubzick C., Ingersoll M.A., Leboeuf M. (2009). Origin of the Lamina Propria Dendritic Cell Network. Immunity.

[18] Greter M., Helft J., Chow A., Hashimoto D., Mortha A., Agudo-Cantero J., Bogunovic M., Gautier E.L., Miller J., Leboeuf M. (2012). GM-CSF Controls Nonlymphoid Tissue Dendritic Cell Homeostasis but Is Dispensable for the Differentiation of Inflammatory Dendritic Cells. Immunity.

[19] King I.L., Kroenke M.A., Segal B.M. (2010). GM-CSF–dependent, CD103+ dermal dendritic cells play a critical role in Th effector cell differentiation after subcutaneous immunization. J. Exp. Med..

[20] Tussiwand R., Everts B., Grajales-Reyes G.E., Kretzer N.M., Iwata A., Bagaitkar J., Wu X., Wong R., Anderson D.A., Murphy T.L. (2015). Klf4 Expression in Conventional Dendritic Cells Is Required for T Helper 2 Cell Responses. Immunity.

[21] Amon L., Seichter A., Vurnek D., Heger L., Lächele L., Tochoedo N.R., Kaszubowski T., Hatscher L., Baranska A., Tchitashvili G. (2024). Clec12A, CD301b, and FcγRIIB/III define the heterogeneity of murine DC2s and DC3s. Cell Rep..

[22] Cossarizza A., Chang H., Radbruch A., Abrignani S., Addo R., Akdis M., Andrä I., Andreata F., Annunziato F., Arranz E. (2021). Guidelines for the use of flow cytometry and cell sorting in immunological studies (third edition). Eur. J. Immunol..

[23] Liu Z., Gu Y., Chakarov S., Bleriot C., Kwok I., Chen X., Shin A., Huang W., Dress R.J., Dutertre C.-A. (2019). Fate Mapping via Ms4a3-Expression History Traces Monocyte-Derived Cells. Cell.

[24] Schlitzer A., Sivakamasundari V., Chen J., Sumatoh H.R.B., Schreuder J., Lum J., Malleret B., Zhang S., Larbi A., Zolezzi F. (2015). Identification of cDC1- and cDC2-committed DC progenitors reveals early lineage priming at the common DC progenitor stage in the bone marrow. Nat. Immunol..

[25] McInnes L., Healy J., Melville J. (2020). UMAP: Uniform Manifold Approximation and Projection for Dimension Reduction. arXiv.

[26] Dress R.J., Dutertre C.-A., Giladi A., Schlitzer A., Low I., Shadan N.B., Tay A., Lum J., Kairi M.F.B.M., Hwang Y.Y. (2019). Plasmacytoid dendritic cells develop from Ly6D+ lymphoid progenitors distinct from the myeloid lineage. Nat. Immunol..

[27] Menezes S., Melandri D., Anselmi G., Perchet T., Loschko J., Dubrot J., Patel R., Gautier E.L., Hugues S., Longhi M.P. (2016). The Heterogeneity of Ly6Chi Monocytes Controls Their Differentiation into iNOS+ Macrophages or Monocyte-Derived Dendritic Cells. Immunity.

[28] Liu Z., Wang H., Li Z., Dress R.J., Zhu Y., Zhang S., De Feo D., Kong W.T., Cai P., Shin A. (2023). Dendritic cell type 3 arises from Ly6C+ monocyte-dendritic cell progenitors. Immunity.

[29] Probst H.C., Stoitzner P., Amon L., Backer R.A., Brand A., Chen J., Clausen B.E., Dieckmann S., Dudziak D., Heger L. (2023). Guidelines for DC preparation and flow cytometry analysis of mouse nonlymphoid tissues. Eur. J. Immunol..

[30] Amon L., Dudziak D., Backer R.A., Clausen B.E., Gmeiner C., Heger L., Jacobi L., Lehmann C.H.K., Probst H.C., Seichter A. (2023). Guidelines for DC preparation and flow cytometry analysis of mouse lymphohematopoietic tissues. Eur. J. Immunol..

[31] Watchmaker P.B., Lahl K., Lee M., Baumjohann D., Morton J., Kim S.J., Zeng R., Dent A., Ansel K.M., Diamond B. (2014). Comparative transcriptional and functional profiling defines conserved programs of intestinal DC differentiation in humans and mice. Nat. Immunol..

[32] Momenilandi M., Lévy R., Sobrino S., Li J., Lagresle-Peyrou C., Esmaeilzadeh H., Fayand A., Floc’h C.L., Guérin A., Della Mina E. (2024). FLT3L governs the development of partially overlapping hematopoietic lineages in humans and mice. Cell.

[33] van Kooyk Y., Ilarregui J.M., van Vliet S.J. (2015). Novel insights into the immunomodulatory role of the dendritic cell and macrophage-expressed C-type lectin MGL. Immunobiology.

[34] Heger L., Balk S., Lühr J.J., Heidkamp G.F., Lehmann C.H.K., Hatscher L., Purbojo A., Hartmann A., Garcia-Martin F., Nishimura S.-I. (2018). CLEC10A Is a Specific Marker for Human CD1c+ Dendritic Cells and Enhances Their Toll-Like Receptor 7/8-Induced Cytokine Secretion. Front. Immunol..

[35] Sallusto F., Lanzavecchia A. (1994). Efficient presentation of soluble antigen by cultured human dendritic cells is maintained by granulocyte/macrophage colony-stimulating factor plus interleukin 4 and downregulated by tumor necrosis factor alpha. J. Exp. Med..

[36] Papaioannou N.E., Salei N., Rambichler S., Ravi K., Popovic J., Küntzel V., Lehmann C.H.K., Fiancette R., Salvermoser J., Gajdasik D.W. (2021). Environmental signals rather than layered ontogeny imprint the function of type 2 conventional dendritic cells in young and adult mice. Nat. Commun..

[37] Rossi D.J., Bryder D., Zahn J.M., Ahlenius H., Sonu R., Wagers A.J., Weissman I.L. (2005). Cell intrinsic alterations underlie hematopoietic stem cell aging. Proc. Natl. Acad. Sci. USA.

[38] Bruno P., Schüler T., Rosshart S.P. (2025). Born to be wild: utilizing natural microbiota for reliable biomedical research. Trends Immunol..

[39] Biburger M., Trenkwald I., Nimmerjahn F. (2015). Three blocks are not enough—Blocking of the murine IgG receptor FcγRIV is crucial for proper characterization of cells by FACS analysis. Eur. J. Immunol..

